# The global burden of polycystic ovary syndrome, endometriosis, uterine fibroids, cervical cancer, uterine cancer, and ovarian cancer from 1990 to 2021

**DOI:** 10.1186/s12889-025-22881-3

**Published:** 2025-05-14

**Authors:** Wei-Zhen Tang, Qin-Yu Cai, Kang-Jin Huang, Wei-Ze Xu, Jia-Zheng Li, Yun-Ren Pan, Hong-Yu Xu, Yi-Fan Zhao, Ting-He Sheng, Zhi-Mou Li, Tai-Hang Liu, Ying-Bo Li

**Affiliations:** 1 Department of Bioinformatics, School of Basic Medical Sciences, Chongqing Medical University, Chongqing, 400016 China; 2 Institute of Neuroscience, Department of Physiology, School of Basic Medical Science, Chongqing Medical University, Chongqing, 400016 China; 3 The Joint International Research Laboratory of Reproduction and Development, Chongqing Medical University, Chongqing, 400016 China; 4 Chongqing Medical University, Box 197, No.1 Yixueyuan Rd, Chongqing, 400016 P. R. China

**Keywords:** Polycystic ovary syndrome, Endometriosis, Uterine fibroids, Cervical cancer, Ovarian cancer, Uterine cancer, Disability-adjusted life-years (DALYs), Burden of disease

## Abstract

**Background:**

Globally, common gynecological disorders such as Polycystic Ovary Syndrome (PCOS), endometriosis, uterine fibroids (non-malignant gynecological diseases), as well as cervical cancer, uterine cancer, and ovarian cancer (gynecological cancers), profoundly impact women’s physical and mental health. The burden of these diseases exhibits significant geographical disparities across different countries and regions, making a comprehensive and precise assessment of the global burden of gynecological diseases particularly crucial. Such an assessment will facilitate the development of region-specific prevention and treatment strategies, contributing to a more effective response to these health challenges.

**Methods:**

Incidence, prevalence, mortality rates, and Disability-Adjusted Life Years (DALYs) data for the aforementioned gynecological conditions were obtained from the 2021 Global Burden of Disease (GBD) study and analyzed by age, location, and year. The burden associated with gynecological diseases was analyzed based on the Socio-demographic Index (SDI) and attributable risk factors. The Estimated Annual Percentage Change (EAPC) and its 95% Confidence Interval (CI) were used to assess temporal trends in burden.

**Results:**

In 2021, uterine fibroids were the leading non-malignant gynecological condition contributing to the highest Age-Standardized Incidence Rate (ASIR) and Age-Standardized Prevalence Rate (ASPR), with rates of 250.93 and 2841.07 per 100,000, respectively. Cervical cancer was the main contributor to the Age-Standardized Mortality Rate (ASMR) and Age-Standardized Disability Rate (ASDR) among the eight selected gynecological diseases, with rates of 6.62 and 226.28 per 100,000, respectively. From 1990 to 2021, the ASIR and ASPR for non-malignant gynecological conditions, such as PCOS and uterine fibroids, increased, while the ASDR for PCOS also rose. Among gynecological cancers, the ASIR for uterine cancer increased, while the ASPR for cervical cancer rose. However, the ASIR for cervical and ovarian cancers decreased, along with reductions in the ASMR and ASDR for these cancers and uterine cancer. There were notable regional disparities based on the SDI. In 2021, lower SDI regions had higher incidence, prevalence, mortality rates, and DALYs for endometriosis and cervical cancer, whereas higher SDI regions saw higher rates for PCOS, uterine fibroids, ovarian cancer, and uterine cancer, with more significant mortality and DALYs for ovarian and uterine cancers. The age distribution of these conditions varied. Non-malignant gynecological conditions, such as PCOS and uterine fibroids, primarily affect women aged 30–34 and 40–69. Endometriosis is most common in women aged 20–34, particularly between 25 and 29. Gynecological cancers, including cervical, uterine, and ovarian cancers, predominantly affect women over 35, especially between 40 and 69, with cervical cancer peaking at ages 50–54. Regarding attributable risk factors globally, 1% of cervical cancer deaths were linked to unsafe sexual behaviors, while a high Body Mass Index(BMI) contributed to 0.09% of ovarian cancer deaths and 0.34% of uterine cancer deaths.

**Conclusion:**

The global burden of these six gynecological conditions poses a significant public health challenge. There is an urgent need for international collaboration to advance the development of age and regionally differentiated management strategies for gynecological diseases, including the development of effective diagnostic screening tools and the implementation of high-quality, targeted prevention and treatment strategies.

**Supplementary Information:**

The online version contains supplementary material available at 10.1186/s12889-025-22881-3.

## Introduction

PCOS, endometriosis, uterine fibroids, cervical cancer, uterine cancer, and ovarian cancer are prevalent gynecological conditions that significantly impact women’s health [1, 2]. These diseases were high prevalence, substantial impact on women’s health, and the considerable burden they place on individuals, healthcare systems, and societies worldwide [3, 4]. With the growing global female population and rapid societal advancement, these diseases not only severely impair the quality of life for patients but may also shorten their life expectancy [5]. Treating and managing these conditions often require substantial resources, imposing a heavy economic burden on patients, families, and society at large. Given the varying burden of these diseases across different regions and populations, a comprehensive and accurate assessment of the global burden of gynecological health is critical.

Gynecological diseases can be categorized into non-malignant conditions and gynecologic cancers. Non-malignant gynecological diseases typically have an insidious onset, high prevalence, and a tendency to recur, affecting women throughout their life cycle. For example, uterine fibroids are the most common benign tumors of the female reproductive system, affecting approximately 20–30% of women [6]. The global prevalence of PCOS is estimated to be around 8–9% [7]. Additionally, endometriosis is associated with about 50% of infertility cases. These diseases, though distinct, share characteristics of recurrence and long-term impact on women’s health [8, 9]. These conditions can lead to a range of severe consequences, including chronic pain, abnormal bleeding, infertility, urinary system issues, and mental health disorders [10–12]. For instance, endometriosis alone accounts for an estimated annual healthcare cost and productivity loss of approximately 11.9 billion dollars in the United States. Given the substantial health costs and economic burden of gynecological conditions, it is particularly important to systematically describe the global burden of benign gynecological diseases. Concurrently, the disease burden of gynecologic cancers varies significantly across different regions and countries. In resource-poor areas, inadequate cancer screening and treatment can lead to increased morbidity and mortality [13]. According to the World Health Organization, cervical cancer is the fourth most common cancer among women globally, with up to 94% of the 350,000 cervical cancer deaths occurring in low- and middle-income countries [14]. In some developing nations, the incidence of cervical cancer continues to rise due to insufficient cervical cancer screening and Human Papillomavirus (HPV) vaccination coverage [15]. Moreover, rapid societal development in developing countries, along with the resulting unhealthy lifestyles and environmental pollution, may also increase the risk of cancer in women. The latest global cancer statistics indicate that the mortality rate of cervical cancer in women from developing countries is significantly higher than that in developed countries, with respective rates of 12.4 and 4.8 per 100,000 [16]. Ovarian cancer poses a significant challenge due to its insidious onset and poor prognosis—often difficult to treat with conventional therapies due to recurrence and drug resistance [17]—while uterine cancer, though more treatable, still ranks as the sixth cause of cancer death in women [18]. Despite their relatively lower incidence, ovarian and uterine cancers remain significant threats to female reproductive system health. Therefore, comprehensive research and analysis of the burden of gynecologic cancers across different regions and countries are crucial for developing more targeted prevention and control strategies.

The GBD 2021 study provides the latest dataset on the burden of non-malignant gynecological diseases and gynecological cancers across 204 countries and territories from 1990 to 2021 [19–21]. Compared to previous GBD studies, this cycle integrated new data sources and improved methodologies to provide updated estimates. This study aims to analyze the trends in incidence, prevalence, mortality, and DALYs of the aforementioned six gynecological conditions from 1990 to 2021. Our goal is to delineate the burden and trends of these diseases at global, regional, and national levels by SDI, age, gender, and associated risk factors, to better understand and address the challenge of gynecological diseases worldwide.

## Method

### Data sources

This study utilized anonymized data from the 2021 GBD Study, a comprehensive database encompassing the impact of 371 diseases, 88 risk factors, and injuries across 204 countries and territories, stratified by five SDI levels [21]. This information is accessible at https://vizhub.healthdata.org/gbd-results and is overseen by the Institute for Health Metrics and Evaluation at the University of Washington [19, 21]. Findings from GBD 2021 are crucial for policymakers, public health professionals, and researchers as they help identify health disparities within and among populations, monitor changes over time, assess progress in health, and devise strategies to address health inequalities post-COVID-19 [20, 21].

In our study, we acquired estimates of incidence, prevalence, mortality, and DALYs for three non-malignant gynecological conditions—PCOS, endometriosis, and uterine fibroids—and three gynecologic cancers—cervical cancer, ovarian cancer, and uterine cancer. Owing to the chronic and non-fatal nature of PCOS, its mortality burden is reported as zero in the GBD study. For GBD 2021, data sources for the three non-malignant gynecological conditions included hospital discharge and claims records, in addition to systematic literature reviews to estimate the prevalence of urolithiasis. Estimates were generated by age, sex, year, and location using a Bayesian meta-regression tool named DisMod-MR 2.1 [21, 22]. Data sources for malignant gynecological cancers included household surveys, censuses, vital statistics, and other health-related data sources. Cause of death ensemble modeling was utilized to estimate mortality rates for specific causes [19, 23]. Disability Weights (DW) represent the severity of health loss or non-fatal disability, with Years Lived with Disability (YLDs) calculated as the number of cases multiplied by the duration until remission or death, and then by the DW [21, 24] Years of Life Lost (YLLs) were determined by multiplying the number of deaths by the standard life expectancy based on age, sex, location, and year. DALYs were computed by adding YLLs to YLDs (with DALYs for PCOS equivalent to YLDs) [24]. The methodology adopted in GBD 2021 closely mirrors that of GBD 2019, with detailed descriptions available elsewhere [25, 26]. The SDI is a composite indicator reflecting the overall development status of a country, considering parameters such as per capita income, average years of education, and fertility rates among young women [21, 23]. The SDI ranges from 0 to 1 and is categorized into quintiles: high (0.805129–1), high-middle (0.689504–0.805129), middle (0.607679–0.689504), low-middle (0.454743–0.607679), and low (0–0.454743) [27].

### Attributable risk factors

The comparative risk assessment framework was developed to generate estimates of the burden of disease attributable to risk factors, which are categorized into four tiers. This study specifically focuses on tier 4 risks [25]. In the 2021 GBD analysis, the process of estimating the burden of risk factors involved seven interconnected methodological steps. The initial assessment included estimating the magnitude of effects due to exposure to identified risk factors by calculating relative risks associated with specific health outcomes. Subsequently, data on exposure were gathered and the distribution across risk factors was evaluated using Bayesian statistical methods. Next, the theoretical minimum risk exposure level (TMREL) was established based on cumulative epidemiological findings. Population Attributable Fractions (PAFs) were then calculated for each risk-outcome pair as an indicator of the potential health improvements that could result if risk exposures were reduced to the TMREL. Age-specific exposure values were computed to reflect the prevalence of exposure after adjusting for age-specific risk factors. Furthermore, mediating factors were estimated to address potential overestimation in PAFs. Finally, attributable burden estimates were determined by multiplying PAF values with the DALYs for each specific age group, sex, geographical location, and calendar year [28].

High BMI was defined as a BMI greater than 20–23 kg/m^2^ in individuals aged 20 years and above. Current smokers were defined as individuals who currently smoke any tobacco product daily or occasionally, while former smokers are those who have ceased using all smoking tobacco products for at least six months. Unsafe sex, including unprotected sexual activity, influenced by alcohol or drugs, refers to engaging in sexual behavior without contraceptive measures or protection against the transmission of sexually transmitted infections, or under impaired mental health. Occupational exposure to asbestos is defined as the percentage of individuals aged 15 years and above with past occupational exposure to asbestos of varying intensities.

### Statistical analysis

Between 1990 and 2021, an analysis of the disease burden for six different gynecological conditions was conducted. This analysis presents all estimated values of disease burden using the number of cases, the ratio of specific age groups, and age-standardized rates (ASRs) per 100,000 population. The ASRs include four different types of age-standardized rates: ASIR, ASPR, ASMR, and ASDR. These indicators are distinct from incidence, prevalence, mortality, and DALYs. ASRs account for the effects of age structure, allowing for fair comparisons of disease burden across different regions or time periods, thereby providing a more accurate reflection of the trends in population health status [29]. In the GBD Study 2021, the ASR was calculated using the formula: ASR = $\:\frac{{\sum\:}_{i=1}^{A}{a}_{i}{w}_{i}}{{\sum\:}_{i=1}^{A}{w}_{i}}\:$ × 100,000, where a_i represents the number of cases in the ith age group, and w_i is the population number (or weight) in the same age group i. The GBD’s standard population structure was used as the reference population to calculate ASRs [21]. All estimates of disease burden were presented as means with 95% uncertainty intervals (UIs). The attributable burden for the three gynecologic cancers was expressed as a percentage of total deaths (%) and a percentage of DALYs (%), each with a 95% UI. The 95% UIs were displayed using the 25th and 975th ranked values from all 1,000 draws. Additionally, we examined the correlation between health metrics for these six gynecological conditions and the SDI. Trends over time were assessed using a linear regression model based on the equation Y = α + βX + ε, where Y is the natural logarithm of the ASR, X represents the calendar year, and ε represents the error term. The EAPC is determined as 100 × [exp(β) − 1] [22, 30], and its 95% confidence interval is obtained from the standard error in the regression model. The EAPC is used to quantify the time trends of different indicators, such as the ASIR, ASPR, ASMR, and ASDR [29]. For each indicator, the regression line is fitted to the natural logarithm of the indicator. Therefore, the EAPC reflects the trend of these indicators over a specific time period. If the EAPC and its 95% confidence interval are both greater than 0, it indicates an increase in the indicator; if both are less than 0, it indicates a decrease; and if the 95% confidence interval includes 0, it suggests that the indicator remained stable with no significant change during the observation period [31]. A List of abbreviations can be found in Table S1.

## Results

### Global incidence, prevalence, mortality and dalys

**Fig. 1:**
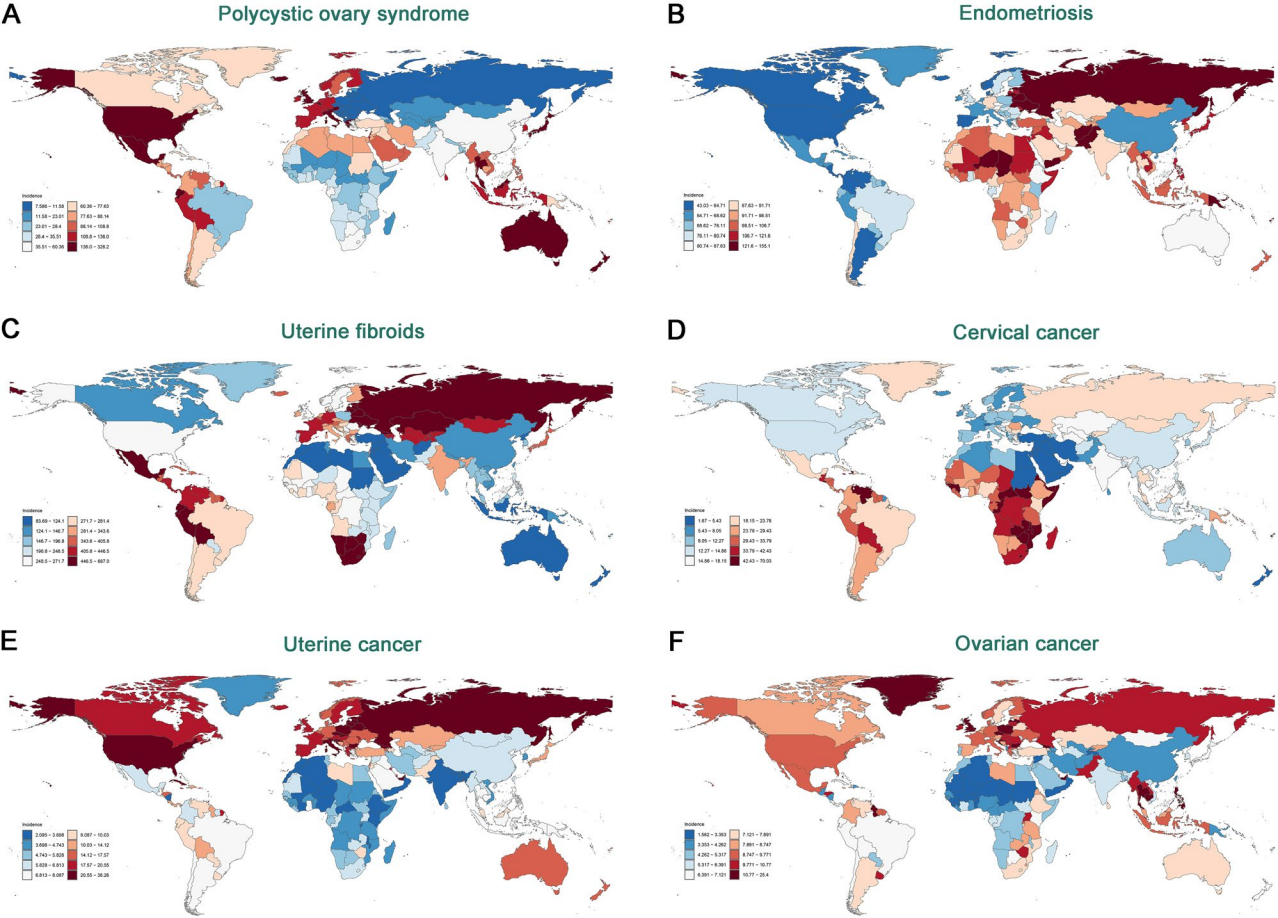
ASIR for six gynecological diseases in 2021: (**A**) PCOS, (**B**) Endometriosis, (**C**) Uterine Fibroids, (**D**) Cervical Cancer, (**E**) Ovarian Cancer, (**F**) Uterine Cancer

**Fig. 2:**
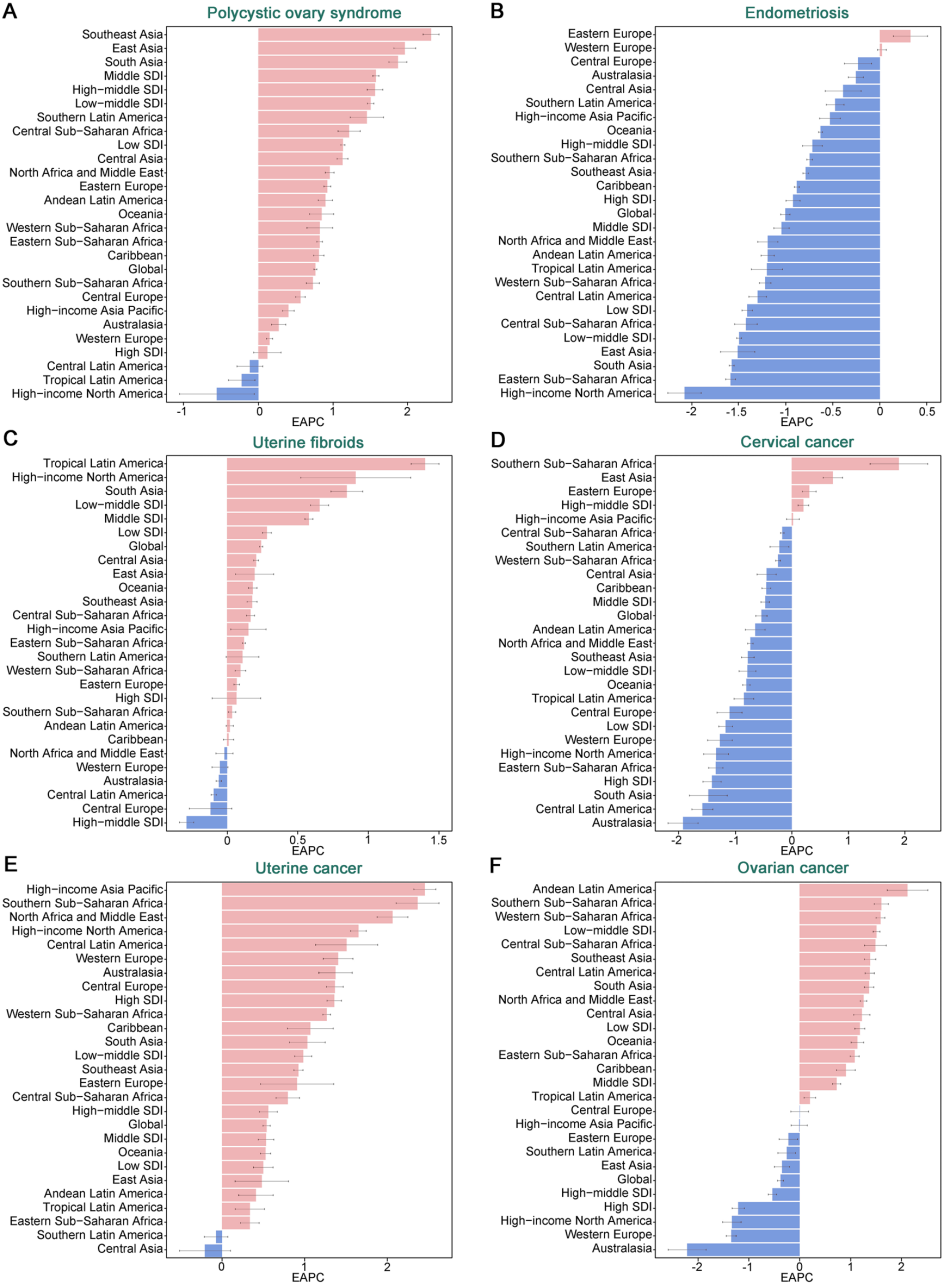
EAPC of ASIR for six gynecological diseases globally and across 21 regions

In 2021, the global incidence of PCOS was 2,302 cases per 100,000 population (95% UI: 1,656-3,167), the incidence of Endometriosis was 3,447 cases per 100,000 population (95% UI: 2,436-4,612), and the incidence of Uterine Fibroids was 10,100 cases per 100,000 population (95% UI: 7,350 − 13,286). Among these non-malignant gynecological diseases, Uterine Fibroids had the highest ASIR at 250.93 cases per 100,000 population (95% UI: 183.44-330.94) (Table 1). From 1990 to 2021, the ASIR of Uterine Fibroids showed an increasing trend, with an EAPC of 0.24% (95% CI: 0.23–0.25%), and the ASIR of PCOS also increased, with an EAPC of 0.77% (95% CI: 0.75–0.79%), while the ASIR of Endometriosis did not show a statistically significant trend (Table 1; Figs. 1 and 2; Table S2). In gynecologic cancers, the global incidences of Cervical Cancer, Ovarian Cancer, and Uterine Cancer in 2021 were estimated at 6.67 × 10^5^ (95% UI: 6.13–7.26), 2.99 × 10^5^ (95% UI: 2.71–3.25), and 4.74 × 10^5^ (95% UI: 4.30–5.14), respectively, with Cervical Cancer having a higher ASIR (15.32 per 100,000 population, 95% UI: 14.08–16.68) compared to Ovarian Cancer (6.71 per 100,000 population, 95% UI: 6.07–7.28) and Uterine Cancer (10.36 per 100,000 population, 95% UI: 9.42–11.24) (Table 1; Fig. 1). From 1990 to 2021, the ASIR of Uterine Cancer showed a significant increasing trend, with an EAPC of 0.54% (95% CI: 0.50–0.58%), while the ASIR of Cervical Cancer showed a decreasing trend, with an EAPC of -0.54% (95% CI: -0.64 to -0.44%) (Table 1; Figs. 1 and 2; Table S3), and the ASIR of Ovarian Cancer did not show a statistically significant trend.

**Table 1:** The global incidence, prevalence, mortality, and dalys for six gynecological system diseases from 1990 to 2021

Year	PCOS	Endometriosis	Uterine fibroids	Cervical cancer	Ovarian cancer	Uterine cancer
1990						
Incidence (× 10^5^, 95% UI)	14.76(10.58–20.45)	33.30(23.09–45.07)	60.10(43.90-80.11)	4.10(3.83–4.39)	1.59(14.57-17,41)	1.91(1.75–2.012)
Prevalence (× 10^5^, 95% UI)	366.51(262.28-506.04)	198.70(135.86–276.90)	656.95(500.22-855.58)	18.26(17.27–19.29)	6.29(5.72–6.93)	13.33(12.26–13.99)
Mortality (95% UI)	-	24.83(13.90-48.88)	1125.85(765.03-1456.02)	211483.79(195724.27-229841.01)	100584.11(92970.81-109087.03)	54848.89(48803.56-59116.13)
DALYs (× 10^5^, 95% UI)	3.24(1.44–6.8)	18.29(10.33–2.86)	0.81(0.57–1.12)	74.16(68.41–80.71)	29.09(26.62–31.99)	15.01(12.98–16.38)
ASIR (1/100,000, 95% UI)	49.45(35.57–68.45)	119.65(83.51-160.46)	234.36(171.06-309.92)	18.11(16.94–19.40)	7.22(6.65–7.87)	8.87(8.12–9.35)
ASPR (1/100,000, 95% UI)	1372.77(984.64-1891.60)	757.10(515.25-1046.13)	2799.88(2133.46-3650.54)	78.15(73.89–82.51)	27.62(25.26–30.26)	61.17(56.35–64.14)
ASMR (1/100,000, 95% UI)	-	0.00(0.00–0.00)	0.05(0.04–0.07)	9.68(8.97–10.51)	4.73(4.38–5.12)	2.60(2.32–2.80)
ASDR (1/100,000, 95% UI)	12.08(5.38–25.21)	69.60(39.69-109.37)	3.48(2.46–4.77)	330.11(304.67–359.10)	132.48(121.34-145.63)	69.17(59.85–75.30)
2021						
Incidence (× 10^5^, 95% UI)	23.02(16.56–31.67)	34.47(24.36–46.12)	101.00(73.50-132.86)	6.67(6.13–7.26)	2.99(2.71–3.25)	4.74(4.30–5.14)
Prevalence (× 10^5^, 95% UI)	694.73(495.31-957.24)	222.75(155.17-304.14)	1195.45(912.28-1549.44)	33.85(31.08–36.97)	12.22(11.02–13.32)	34.52(31.65–37.25)
Mortality (95% UI)	-	54.17(21.51-121.92)	2077.75(1225.41-2574.28)	296667.24(272058.62-321905.72)	185608.68(167961.98-201012.67)	97672.08(86515.79-108061.54)
DALYs (× 105, 95% UI)	6.08(2.73–12.69)	20.49(11.95–31.34)	1.43(1.02–1.93)	99.12(90.53-107.98)	51.63(46.92–56.08)	25.63(22.91–28.46)
ASIR (1/100,000, 95% UI)	63.26(45.41–87.28)	88.52(62.53-119.55)	250.93(183.44-330.94)	15.32(14.08–16.68)	6.71(6.07–7.28)	10.36(9.42–11.24)
ASPR (1/100,000, 95% UI)	1757.83(1253.36-2421.26)	556.98(388.85-764.05)	2841.07(2164.43-3682.27)	79.30(72.81–86.58)	28.08(25.26–30.64)	75.73(69.37–81.78)
ASMR (1/100,000, 95% UI)	-	0.00(0.00–0.00)	0.05(0.03–0.06)	6.62(6.07–7.18)	4.06(3.67–4.40)	2.11(1.87–2.34)
ASDR (1/100,000, 95% UI)	15.40(6.91–32.13)	51.27(29.87–78.43)	3.39(2.43–4.59)	226.28(206.51-246.86)	115.15(104.58-125.21)	56.15(50.07–62.37)
1990–2021						
ASIR (EAPC, 95% CI)	0.77(0.75–0.79)	-1.00(-1.05–0.95)	0.24(0.23–0.25)	-0.54(-0.64–0.44)	-0.38(-0.43–0.32)	0.54(0.50–0.58)
ASPR (EAPC, 95% CI)	0.74(0.70–0.77)	-1.02(-1.07–0.97)	0.04(0.03–0.06)	0.12(0.03–0.21)	-0.07(-0.13-0.00)	0.77(0.72–0.82)
ASMR (EAPC, 95% CI)	-	1.27(1.03–1.51)	0.06(-0.07-0.20)	-1.27(-1.36–1.18)	-0.62(-0.68–0.57)	-0.78(-0.85–0.70)
ASDR (EAPC, 95% CI)	0.72(0.68–0.76)	-1.01(-1.06–0.96)	0.05(-0.01-0.11)	-1.27(-1.36–1.17)	-0.59(-0.64–0.54)	-0.78(-0.85–0.71)

In 2021, the global prevalence of Uterine Fibroids was 11,954.5 cases per 100,000 population (95% UI: 9,122.8–15,494.4), with an ASPR of 2,841.07 cases per 100,000 population (95% UI: 2,164.43-3,682.27), exceeding the prevalence of PCOS and Endometriosis (Table 1; Figures S1; S4). From 1990 to 2021, the ASPR of both PCOS and Uterine Fibroids showed a significant upward trend (PCOS, EAPC = 0.74%, 95% CI: 0.70–0.77%; Uterine Fibroids, EAPC = 0.04%, 95% CI: 0.03–0.06%), while the ASPR of Endometriosis showed a downward trend (EAPC = -1.02%, 95% CI: -1.07 to -0.97%) (Table 1; Table S2; Figures S1; S4). In the same year, the global prevalence and ASPR of Uterine Cancer and Cervical Cancer exceeded those of Ovarian Cancer; from 1990 to 2021, the ASPR of both Uterine Cancer and Cervical Cancer showed a significant increasing trend (Uterine Cancer, EAPC = 0.77%, 95% CI: 0.72–0.82%; Cervical Cancer, EAPC = 0.12%, 95% CI: 0.03–0.21%), while the ASPR of Ovarian Cancer showed a significant decreasing trend (EAPC = -0.07%, 95% CI: -0.13 to 0.00%) (Table 1; Table S3; Figures S1; S4).

In 2021, the global number of deaths attributed to Uterine Fibroids was estimated to be 2,077.75 cases (95% UI: 1,225.41-2,574.28), and the number of deaths attributed to Endometriosis was approximately 54.17 cases (95% UI: 21.51-121.92) (Table 1; Figure S2). The ASMR for Uterine Fibroids showed an increasing trend (EAPC = 0.05%, 95% CI: 0.03–0.06%) (Table 1; Table S2; Figure S2; S5). The ASMR for Cervical Cancer (6.62, 95% UI: 6.07–7.18) was significantly higher than the other two cancers. However, from 1990 to 2021, the ASMR for all three cancers showed a decreasing trend, with the EAPC for Cervical Cancer being − 1.27% (95% CI: -1.36 to -1.18%), Ovarian Cancer being − 0.62% (95% CI: -0.68 to -0.57%), and Uterine Cancer being − 0.78% (95% CI: -0.85 to -0.70%) (Table 1; Table S3; Figure S2; S5).

In 2021, Endometriosis led to the highest global burden of disease (DALYs) among the three gynecological benign diseases, with 2,049 cases per 100,000 population (95% UI: 1,195-3,134) (Table 1; Figure S3), and the highest EAPC for ASDR at 51.27% (95% CI: 29.87–78.43%) (Table 1; Table S2; Figure S6). Among gynecologic cancers, Cervical Cancer had the highest ASDR, with 226.28 cases per 100,000 population (95% UI: 206.51-246.53). From 1990 to 2021, the ASDR for all three cancers showed a decreasing trend, with the most significant reduction for Cervical Cancer (EAPC = -1.27%, 95% CI: -1.36 to -1.17%) (Table 1; Table S3; Figure S6).

### Regional incidence, prevalence, mortality, and dalys

In 2021, South Asia led globally in incidence, prevalence, mortality, and DALYs for PCOS, Endometriosis, and Uterine Fibroids. Specifically, South Asia had the highest incidence rates of PCOS (4.16 × 10^5^, 95% UI: 3.00–5.74), Endometriosis (9.37 × 10^5^, 95% UI: 6.43–12.69), and Uterine Fibroids (29.03 × 10^5, 95% UI: 20.98–38.95) (Table 2). Additionally, this region reported the highest prevalence figures for PCOS (112.91 × 10^5^, 95% UI: 79.50–158.33), Endometriosis (58.66 × 10^5^, 95% UI: 40.81–81.59), and Uterine Fibroids (310.26 × 10^5^, 95% UI: 231.12–412.27). South Asia also recorded the highest number of Uterine Fibroid-related deaths (632.86, 95% UI: 382.44–987.05), while Southeast Asia had the highest number of Endometriosis-related deaths (14.45, 95% UI: 4.92–34.37) (Tables S3, S4). Furthermore, South Asia had the highest global DALYs for PCOS (0.99 × 10^5^, 95% UI: 0.43–2.07), Endometriosis (5.37 × 10^5^, 95% UI: 3.13–8.30), and Uterine Fibroids (0.49 × 10^5^, 95% UI: 0.35–0.68) (Table S6; Figs. 1; S1-3).

**Table 2:** The regional incidence and ASIR of six gynecological diseases in 2021

Location	PCOS		Endometriosis		Uterine fibroids		Cervical cancer		Ovarian cancer		Uterine cancer	
	Incidence (× 105, 95% UI)	ASIR (1/100,000, 95% UI)	Incidence (× 105, 95% UI)	ASIR (1/100,000, 95% UI)	Incidence (× 105, 95% UI)	ASIR (1/100,000, 95% UI)	Incidence (× 105, 95% UI)	ASIR (1/100,000, 95% UI)	Incidence (× 105, 95% UI)	ASIR (1/100,000, 95% UI)	Incidence (× 105, 95% UI)	ASIR (1/100,000, 95% UI)
Global	23.02(16.56–31.67)	63.26(45.41–87.28)	34.47(24.36–46.12)	88.52(62.53-119.55)	101.00(73.50–132,86)	250.93(183.44-330.94)	6.67(6.13–7.26)	15.32(14.08–16.68)	2.99(2.71–3.25)	6.71(6.07–7.28)	4.74(4.30–5.14)	10.36(9.42–11.24)
High SDI	4.94(3.67–6.71)	144.89(107.28-196.81)	3.58(2.61–4.67)	75.36(54.15–99.16)	14.10(10.17–18.38)	265.52(193.64-348.39)	0.78(0.74–0.81)	10.30(9.91–10.66)	0.81(0.73–0.85)	8.40(7.84–8.81)	1.92(1.77-2.00)	19.55(18.28–20.35)
High-middle SDI	3.06(2.16–4.25)	72.52(51.14-101.53)	4.89(3.53-6,48)	83.07(59.41-110.49)	17.71(12.94–23.11)	256.86(188.01-334.87)	1.20(1.04–1.38)	13.27(11.44–15.16)	0.68(0.60–0.76)	6.93(6.10–7.76)	1.48(1.35–1.64)	14.23(12.89–15.75)
Middle SDI	8.13(5.73–11.26)	77.38(54.44-107.63)	10.08(7.09–13.49)	82.40(58.07–111.20)	31.20(22.74–41.03)	238.21(174.87-313.82)	2.28(2.05–2.54)	15.94(14.30-17.75)	0.82(0.72–0.92)	5.81(5.07–6.50)	0.90(0.73.95–1.06)	6.09(4.97–7.20)
Low-middle SDI	4.82(3.38–6.70)	45.20(31.82–62.95)	9.82(6.81–13.37)	94.13(65.77-127.29)	26.09(19.06–34.90)	262.69(191.79-350.05)	1.53(1.37–1.69)	17.79(15.94–19.70)	0.50(0.43–0.59)	6.01(5.22–7.07)	0.33(0.28–0.40)	4.13(3.56–5.08)
Low SDI	2.05(1.44–2.91)	27.79(19.81–38.87)	6.08(4.20–8.49)	103.56(72.04-141.06)	11.83(8.59–15.88)	237.87(171.82-315.92)	0.88(0.74–1.04)	25.47(21.57–30.12)	0.17(0.13–0.21)	5.42(4.12–6.43)	0.10(0.08–0.13)	3.58(2.87–4.59)
Andean Latin America	0.43(0.29–0.60)	134.14(92.65–190.70)	0.25(0.17–0.34)	70.91(49.07–97.55)	1.86(1.36–2.47)	521.33(380.49-694.99)	0.10(0.07–0.12)	29.79(22.83–37.66)	0.02(0.01–0.03)	6.90(5.27–8.68)	0.03(0.02–0.04)	9.97(7.40–12.90)
Australasia	0.21(0.15–0.30)	202.25(145.07-278.86)	0.12(0.08–0.16)	88.39(61.29-119.58)	0.14(0.10–0.19)	87.86(63.79-117.13)	0.02(0.01–0.02)	8.51(7.66–9.35)	0.02(0.01-0,02)	7.18(6.51–7.81)	0.04(0.03–0.04)	14.65(12.84–16.34)
Caribbean	0.12(0.08–0.17)	55.61(37.61–78.19)	0.17(0.12–0.23)	70.85(48.81–97.49)	0.90(0.65–1.22)	367.58(263.71-497.19)	0.07(0.06–0.09)	27.58(23.09–32.63)	0.05(0.04–0.06)	6.66(5.70–8.07)	0.02(0.01–0.02)	17.48(15.17–20.10)
Central Asia	0.08(0.06–0.11)	18.79(13.06–26.29)	0.42(0.29–0.56)	89.03(61.79-118.98)	2.26(1.62–2.96)	436.75(315.72–568.20)	0.07(0.06–0.08)	14.17(12.38–15.92)	0.03(0.02–0.03)	6.10(5.31–6.94)	0.05(0.04–0.05)	9.71(8.60-10.95)
Central Europe	0.03(0.02–0.04)	8.97(6.22–12.67)	0.36(0.26–0.49)	76.29(53.69-102.95)	1.52(1.11–1.97)	248.04(185.24-316.71)	0.14(0.13–0.16)	15.93(14.45–17.53)	0.11(0.11–0.12)	10.80(9.93–11.68)	0.23(0.21–0.25)	20.70(18.66–22.70)
Central Latin America	1.43(1.01-2.00)	115.89(81.09-162.25)	0.90(0.62–1.22)	65.31(45.28–88.82)	6.21(4.45–8.16)	446.82(319.89-588.04)	0.40(0.35–0.46)	28.89(24.76–33.10)	0.11(0.10–0.13)	8.23(7.16–9.29)	0.10(0.09–0.11)	7.08(6.19–8.06)
Central Sub-Saharan Africa	0.24(0.17–0.34)	26.20(18.46–36.97)	0.69(0.47–0.97)	99.03(67.59–135.70)	1.59(1.17–2.11)	267.88(195.21-353.82)	0.15(0.11–0.21)	38.00(26.28–50.98)	0.02(0.01–0.02)	4.38(2.49–6.25)	0.01(0.01–0.02)	4.07(2.64–5.92)
East Asia	2.68(1.90–3.74)	58.63(41.20-82.32)	4,45(3.20–5.93)	66.99(47.90-89.29)	10.16(7.35–13.25)	136.50(101.31-177.06)	1.38(1.01–1.78)	13.40(9.86–17.38)	0.44(0.33–0.57)	4.21(3.15–5.46)	0.76(0.57–1.03)	6.73(5.02–9.23)
Eastern Europe	0.08(0.05–1.06)	10.86(7.64–15.38)	1.18(0.84–1.57)	133.62(93.16-180.27)	7.05(5.01–9.32)	610.42(445.44-798.58)	0.25(0.23–0.28)	16.49(14.80–18.10)	0.18(0.16–0.20)	9.83(8.77–11.03)	0.59(0.53–0.65)	29.70(26.77–32.90)
Eastern Sub-Saharan Africa	0.77(0.54–1.09)	26.26(18.57–36.96)	1.96(1.35–2.73)	85.16(58.63-116.99)	3.98(2.86–5.37)	206.20(149.22-275.53)	0.41(0.33–0.53)	33.45(27.17–42.12)	0.09(0.06–0.11)	4.10(3.00-5.59)	0.04(0.03–0.05)	7.48(5.31–9.25)
High-income Asia Pacific	1.07(0.76–1.52)	225.11(160.59-317.34)	0.77(0.55–1.03)	104.40(73.38-137.09)	2.17(1.60–2.82)	296.79(219.27-386.14)	0.16(0,14-0.17)	11.03(10.25–11.95)	0.11(0.10–0.12)	6.36(5.69–6.75)	0.14(0.12–0.15)	8.31(7.57–9.04)
High-income North America	2.03(1.50–2.71)	149.79(111.27-197.64)	0.91(0.65–1.19)	54.13(38.61–71.05)	4.51(3.20–5.94)	252.30(180.16-335.13)	0.30(0.29–0.32)	12.69(12.19–13.22)	0.28(0.26–0.29)	8.88(8.29–9.27)	1.04(0.96–1.09)	31.78(29.88–33.21)
North Africa and Middle East	2.42(1.71–3.43)	77.15(54.45-109.17)	3.41(2.39–4.62)	106.65(74.66-144.42)	4.02(2.90–5.40)	122.58(88.51-163.47)	0.13(0.11–0.15)	4.72(4.04–5.50)	0.12(0.10–0.14)	4.87(3.96–5.63)	0.17(0.13–0.21)	7.07(5.17–8.40)
Oceania	0.05(0.04–0.07)	69.33(48.42–97.52)	0.11(0.07–0.15)	148.30(104.46-204.29)	0.10(0.07–0.13)	139.06(101.13-189.15)	0.01(0.01–0.02)	27.31(21.47–39.68)	0.00(0.00–0.00)	3.90(2.32–5.28)	0.00(0.00-0.01)	8.19(4.75–12.27)
South Asia	4.16(3.00-5.74)	42.08(29.94–58.32)	9.37(6.43–12.69)	92.24(63.83-124.65)	29,03(20.98–38.95)	296.47(213.91-395.25)	1.32(1.15–1.51)	15.54(13.47–17.71)	0.50(0.42–0.59)	5.99(5.13–7.14)	0.24(0.20–0.32)	2.99(2.50–3.97)
Southeast Asia	3.57(2.54–4.95)	110.11(77.87-153.89)	3.86(2.72–5.18)	104.98(73.69–141.20)	5.43(3.99–7.18)	144.48(106.40-191.70)	0.58(0.49–0.68)	15.17(12.91–17.65)	0.35(0.26–0.46)	9.28(6.97–12.04)	0.25(0.17–0.31)	6.55(4.56–7.99)
Southern Latin America	0.22(0.15–0.31)	74.53(52.77-106.57)	0.25(0.174–0.32)	69.91(49.38–91.46)	1.01(0.71–1.35)	276.55(193.62-371.61)	0.09(0.09–0.10)	22.80(21.07–24.73)	0.03(0.03–0.04)	7.69(7.14–8.23)	0.03(0.03–0.04)	7.20(6.50–7.96)
Southern Sub-Saharan Africa	0.18(0.13–0.26)	42.33(29.66–59.97)	0.41(0.28–0.55)	92.10(63.35-125.33)	2.40(1.75–3.27)	534.10(390.68-720.81)	0.16(0.14–0.18)	42.40(37.16–47.85)	0.03(0.02–0.03)	7.71(5.88–8.93)	0.02(0.02–0.03)	6.47(4.86–7.56)
Tropical Latin America	0.24(0.17–0.33)	24.90(17.12–35.01)	0.91(0.63–1.20)	75.98(52.64-102.04)	3.64(2.65–4.763)	278.54(204.44-365.57)	0.28(0.26–0.29)	20.27(19.16–21.27)	0.09(0.08–0.09)	6.43(6.05–6.77)	0.10(0.96 − 0.11)	7.27(6.74–7.72)
Western Europe	2.06(1.46–2.87)	154.64(109.19-215.84)	1.26(0.90–1.67)	72.45(50.52–97.95)	7.11(5.13–9.37)	345.03(250.25-460.96)	0.28(0.26–0.29)	8.71(8.25–9.11)	0.38(0.34–0.41)	8.99(8.35–9.44)	0.81(0.73–0.86)	18.48(17.01–19.49)
Western Sub-Saharan Africa	0.93(0.65–1.33)	27.80(19.81–39.19)	2.72(1.87–3.81)	105.39(72.72-142.49)	5.90(4.30–7.97)	272.75(198.05–364.30)	0.35(0.27–0.43)	24.11(18.93–29.10)	0.05(0.03–0.06)	3.62(2.40–4.64)	0.04(0.03–0.05)	3.55(2.73–4.50)

**Fig. 3:**
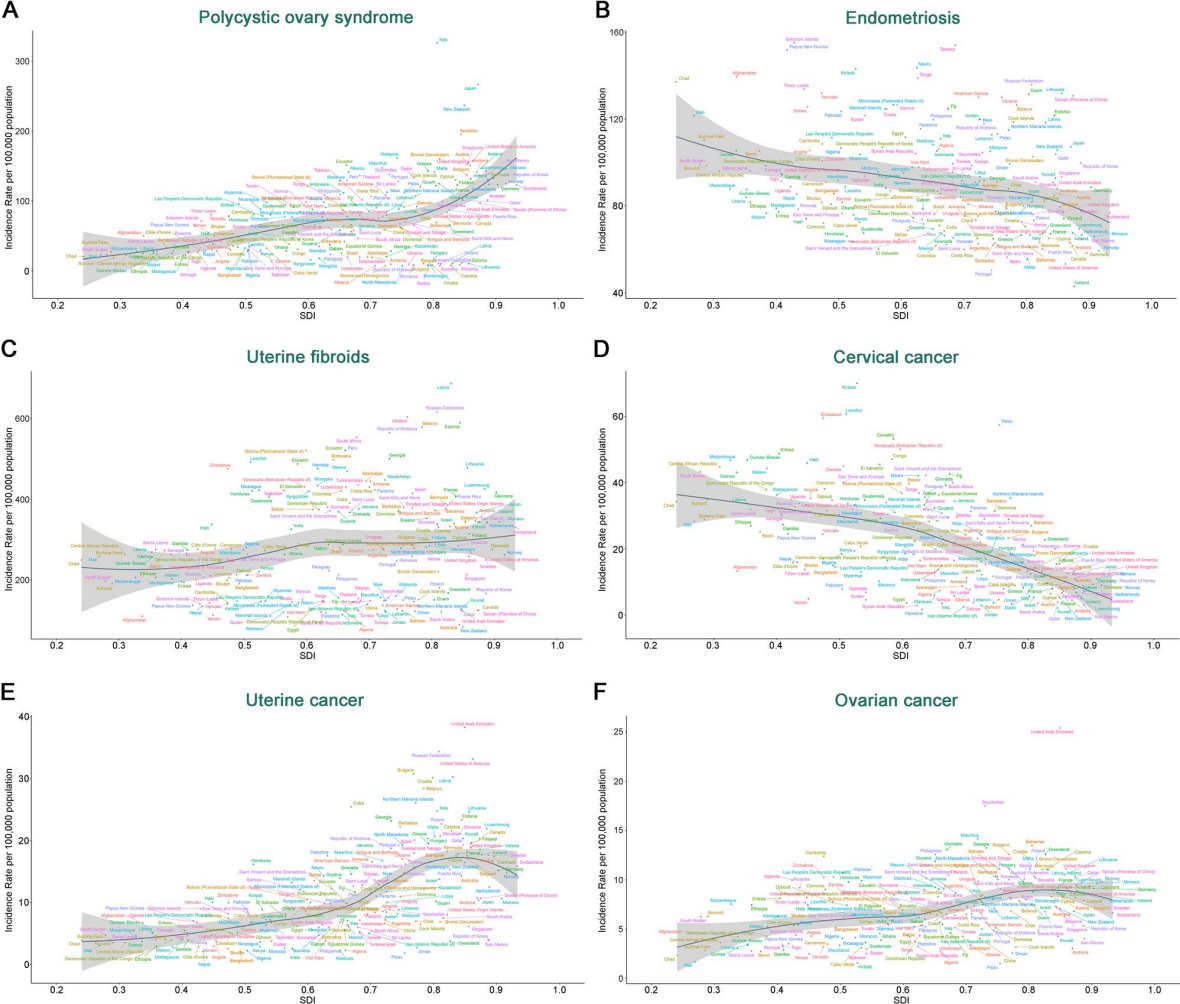
SDI and its association with ASIR for six gynecological diseases across 204 countries and territories

In 2021, the High-income Asia Pacific region had the highest ASIR for PCOS at 225.11 per 100,000 (95% UI: 160.59–317.34), the highest ASPR at 5,237.62 per 100,000 (95% UI: 3,779.21–7,307.15), and the highest ASDR at 45.62 per 100,000 (95% UI: 20.81–92.93). In contrast, Oceania reported the highest age-standardized rates for Endometriosis: incidence (148.30 per 100,000, 95% UI: 104.46–204.29), prevalence (996.78 per 100,000, 95% UI: 673.44–1,378.56), and DALYs (91.69 per 100,000, 95% UI: 52.17–145.64). Eastern Europe exhibited the highest age-standardized incidence (610.42 per 100,000, 95% UI: 445.44–798.58) and prevalence rates (6,934.45 per 100,000, 95% UI: 5,230.48–8,997.04) for Uterine Fibroids; however, Southern Sub-Saharan Africa had the highest age-standardized DALY rate (10.58 per 100,000, 95% UI: 7.30–13.62) (Table 2; Tables S3–S5). From 1990 to 2021, Southeast Asia experienced the most significant increase in the burden of PCOS. The greatest increases in the burden of Endometriosis were observed in Eastern Europe and East Asia, while Tropical Latin America and East Asia saw the highest growth in the burden of Uterine Fibroids (Tables S3–S5; Fig. 2; Figures S4–S6).

In 2021, East Asia had the highest incidence (1.38 × 10^5^, 95% UI: 1.01–1.78) and prevalence (7.75 × 10^5^, 95% UI: 5.62–10.10) rates of Cervical Cancer, while South Asia had the highest mortality (0.70 × 10^5, 95% UI: 0.61–0.80) and DALYs (24.32 × 10^5, 95% UI: 20.98–27.73). For Ovarian Cancer, South Asia recorded the highest incidence (0.50 × 10^5^, 95% UI: 0.42–0.59), prevalence (1.93 × 10^5^, 95% UI: 1.61–2.31), mortality (0.31 × 10^5, 95% UI: 0.26–0.36), and DALYs (9.60 × 10^5^, 95% UI: 8.23–11.42). Regarding Uterine Cancer, the High-income North America region showed the highest incidence (1.04 × 10^5^, 95% UI: 0.96–1.09) and prevalence rates (8.09 × 10^5^, 95% UI: 7.57–8.47), while Western Europe had the highest mortality (0.14 × 10^5^, 95% UI: 0.12–0.15) and East Asia the highest DALYs (4.25 × 10^5^, 95% UI: 3.20–5.71) (Table 2; Tables S3–S5). Notably, from 1990 to 2021, Southern Sub-Saharan Africa experienced rapid increases in incidence, mortality, and DALYs for Cervical Cancer, while East Asia showed significant increases in incidence (Tables S3–S5). From 1990 to 2021, most regions saw an increase in the burden of Ovarian Cancer, particularly in Andean Latin America. For Uterine Cancer, the High-income Asia Pacific had the fastest growth in incidence and prevalence, while Southern Sub-Saharan Africa saw the quickest increase in mortality and DALYs (Fig. 2; Figures S4-6).

**Fig. 4:**
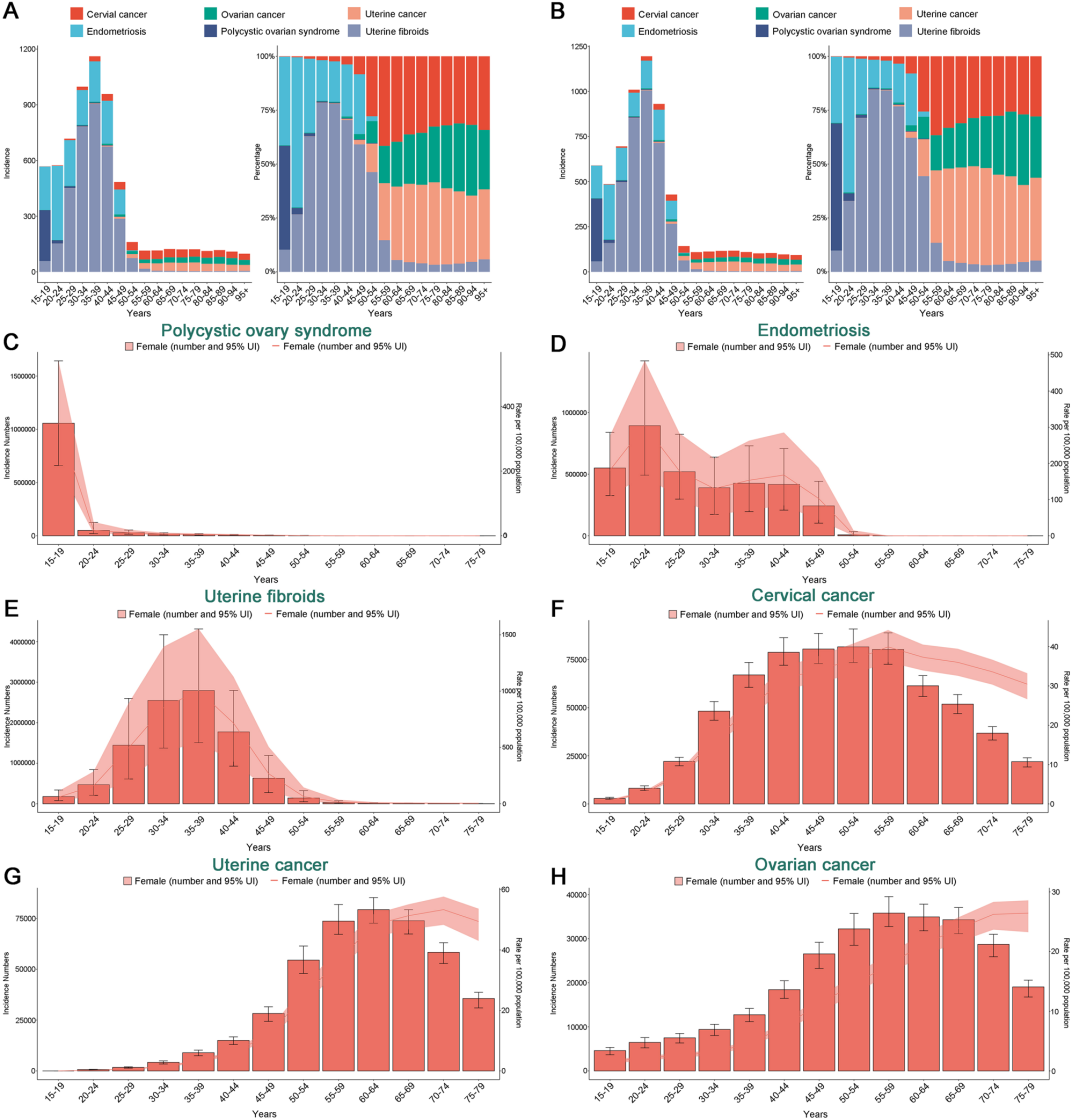
Incidence rates of gynecological diseases by different age-etiologic compositions in 1990 and 2021, and the global incidence rates of six gynecological diseases by age in 2021

### The burden of six gynecological diseases in relation to the SDI

In 2021, varying levels of the SDI significantly influenced the epidemiological characteristics of both benign and malignant gynecological diseases. At lower and middle SDI quintiles, endometriosis and uterine fibroids, both benign gynecological diseases, showed higher incidence, prevalence, mortality, and DALYs, reflecting a greater burden in regions with lower socioeconomic development. In contrast, at higher SDI quintiles, PCOS and gynecological cancers such as cervical cancer, uterine cancer, and ovarian cancer demonstrated higher prevalence, indicating their greater occurrence in more developed regions. Interestingly, while uterine fibroids, a benign gynecological disease, showed higher incidence and prevalence in higher SDI regions, it was associated with higher DALYs and mortality in lower SDI regions. This contrast highlights the different burden patterns between benign and malignant diseases, underlining the influence of economic development and healthcare infrastructure on the epidemiological trends of both disease types(Table 2; Figures S7–S10).

Across 204 countries and territories, the ASIR and ASPR for PCOS, Uterine Fibroids, Ovarian Cancer, and Uterine Cancer were positively correlated with SDI, with the ASDR for PCOS and Ovarian Cancer also showing a positive correlation with SDI. Additionally, the ASMR for Ovarian Cancer was positively correlated with SDI. However, we observed a negative correlation between SDI and the ASIR, ASPR, ASMR, and ASDR for both Endometriosis and Cervical Cancer. The ASDR and ASMR for Uterine Fibroids also showed a negative correlation with SDI. No significant association was found between the ASMR or ASDR for Uterine Cancer and SDI (Fig. 3; Figures S11–S13).

### The burden of six gynecological diseases stratified by age

In 2021, six major gynecological diseases exhibited distinct patterns of incidence across different female age groups. For non-malignant gynecological diseases, the incidence of PCOS peaked during late adolescence (ages 15–19), with the highest concentration of prevalence and DALYs in women aged 20 to 34, particularly between 30 and 34 years, where prevalence reached its apex (Fig. 4; Figures S14–S16). Endometriosis showed a different pattern, with its incidence, prevalence, and DALYs primarily concentrated in women aged 20 to 34, with the highest incidence rate occurring in the 20 to 24 age group, and the highest prevalence and DALYs in the 25 to 29 age bracket. Notably, the highest mortality rate was observed in women aged 45 to 49. Uterine fibroids predominantly affected women aged 35 to 49, with the highest incidence rates, prevalence, and DALYs within this age range; the incidence was most pronounced in the 35–39 age group, while the 40–44 age cohort experienced the most severe prevalence and DALYs. Mortality rates were more common in individuals over 40, particularly peaking at ages 45 to 49 (Fig. 4; Figures S14–S16).

For gynecological cancers, the incidence, prevalence, and DALYs of cervical cancer were mainly concentrated in the 40–59 age group, with the highest incidence and DALYs observed in the 50 to 54 age bracket, the highest prevalence at ages 40 to 44, and the greatest mortality rate in the 55 to 59 age range. Ovarian cancer’s incidence, prevalence, and DALYs were primarily focused in the 50–69 age group, with the 55 to 59 age bracket experiencing peak rates of incidence, prevalence, and DALYs, and the highest mortality rate occurring in the 65 to 69 demographic. Lastly, the incidence, prevalence, and DALYs of uterine cancer were mainly concentrated in the 55–69 age group, with the highest incidence and prevalence in the 60 to 64 age bracket, the greatest DALYs in the 65 to 69 age range, and mortality also being most common in individuals aged 65–69.

### Attributable burden of gynecological cancers due to risk factors

In our study, it was found that a high BMI is a risk factor for uterine cancer. Additionally, unsafe sex and smoking were identified as risk factors for cervical cancer. Moreover, ovarian cancer was found to be associated with a high body-mass index and occupational exposure to asbestos.

Data from 2021 indicate that unsafe sexual practices were a significant contributor to cervical cancer mortality and DALYs, accounting for 1.00% (95% CI 0.99–1.00) of cervical cancer-related deaths and 1.00% (95% CI 1.00–1.00) of DALYs globally (Table 3). Additionally, smoking, as another contributing factor, was responsible for 0.08% (95% CI 0.04–0.11) of cervical cancer deaths and 0.07% (95% CI 0.04–0.10) of DALYs worldwide (Table 3).

**Table 3:** Percentage of gynecological cancer deaths and dalys attributable to risk factors in 1990 and 2021

Cancer	1990			2021		
	Leading risk	Percentage of death (95% UI)	Percentage of DALYs (95% UI)	Leading risk	Percentage of death (95% UI)	Percentage of DALYs (95% UI)
Cervical cancer	Unsafe sex	1.00(0.99,1.00)	1.00(0.99,1.00)	Unsafe sex	1.00(0.99,1.00)	1.00(1.00,1.00)
	Smoking	0.11(0.07,0.15)	0.11(0.06,0.15)	Smoking	0.08(0.04,0.11)	0.07(0.04,0.10)
Ovarian cancer	High body-mass index	0.07(0,01,0.13)	0.07(0,01,0.12)	High body-mass index	0.09(0.02,0.17)	0.09(0.02,0.16)
	Occupational exposure to asbestos	0.04((0.02,0.06)	0.03(0.01,0.04)	Occupational exposure to asbestos	0.03(0.01,0.05)	0.02(0.01,0.03)
Uterine cancer	High body-mass index	0.25(0.18–0.33)	0.25(0.18–0.33)	High body-mass index	0.34(0.25,0.44)	0.34(0.25,0.44)

For ovarian cancer, a high BMI was identified as a significant risk factor, leading to 0.09% (95% UI 0.02–0.17) of ovarian cancer deaths and 0.09% (95% UI 0.02–0.16) of DALYs globally. Furthermore, occupational asbestos exposure was also considered a risk factor, albeit with a relatively minor impact, causing 0.03% (95% CI 0.01–0.05) of ovarian cancer deaths and 0.02% (95% CI 0.01–0.03) of DALYs worldwide (Table 3). In the case of uterine cancer, a high body-mass index remained a principal risk factor, with data from 2021 showing that high BMI contributed to 0.34% (95% CI 0.25–0.44) of uterine cancer deaths and 0.34% (95% UI 0.25–0.44) of DALYs globally (Table 3).

## Discussion

As global awareness of the significant threat posed by gynecological diseases to global health deepens, the increasing prevalence of these conditions presents growing challenges to public health policies and healthcare systems worldwide. The far-reaching impact of gynecological diseases necessitates global attention and immediate action. This study provides a comprehensive assessment of the burden of gynecological diseases at global, regional, and national levels from 1990 to 2021, stratified by SDI and age groups. In 2021, uterine fibroids exhibited the highest ASIR and ASPR, while cervical cancer had the highest ASMR and DALY rate. From 1990 to 2021, non-malignant gynecological conditions such as PCOS and uterine fibroids showed an upward trend in both ASIR and ASPR, with the ASMR for PCOS also increasing. For gynecological cancers, the ASIR for uterine cancer increased, and the ASPR for cervical cancer also rose. Concurrently, the ASIR for cervical and ovarian cancers displayed a downward trend, along with a decrease in ASMR and ASDR for uterine cancer. Additionally, regions in the lower and middle SDI quintiles suffered higher incidence, prevalence, mortality rates, and DALYs from endometriosis and cervical cancer. In contrast, regions in the highest SDI quintile in 2021 had higher incidence and prevalence rates for PCOS, uterine fibroids, ovarian cancer, and uterine cancer, with mortality rates and DALYs also higher for ovarian and uterine cancers. Accurate epidemiological data on gynecological diseases are crucial for the equitable allocation of limited resources at global, regional, and national levels. Building on previous research, this study offers a comprehensive evaluation of the global burden of gynecological diseases, laying a solid foundation for healthcare service planning and intervention programs.

The study demonstrates that the SDI is associated with the incidence, prevalence, mortality rates, and DALYs for six gynecological conditions. Data from 2021 across 204 countries and territories showed a positive correlation between SDI and the ASIR and ASPR for PCOS, uterine fibroids, ovarian cancer, and uterine cancer. Furthermore, the ASDR for PCOS and ovarian cancer, as well as the ASMR for ovarian cancer, also showed a positive correlation with SDI. However, a negative correlation was observed between SDI and the ASIR, ASPR, ASMR, and ASDR for endometriosis and cervical cancer. Notably, the ASDR and ASMR for uterine fibroids showed a negative correlation with SDI. For uterine cancer, no significant correlation was found between its ASMR, ASDR, and SDI.

In non-malignant gynecological diseases, the positive correlation between SDI and the burden of PCOS may be due to higher adherence to Westernized diets in developed countries, which are associated with increased risks of obesity, insulin resistance, and precocious puberty, all of which are related to the development of PCOS [32, 33]. Additionally, limitations in screening, treatment, and disease management services in developing countries may lead to an underestimation of PCOS, thereby precipitating long-term complications and comorbidities, imposing significant burdens on individuals and societies [34]. Conversely, an overestimation of PCOS may occur in countries with more accessible healthcare services, possibly resulting in unnecessary exposure to drug-related side effects and potential hindrance to future medical insurance access. For endometriosis, regions may show a negative correlation with SDI due to more comprehensive disease management and preventive measures, including stricter control of disease burden and higher quality medical care. Rapidly developing societies typically boast more robust healthcare systems, such as widespread use of assisted reproductive technologies or laparoscopic techniques, which may reduce the likelihood of infertility becoming a major issue. However, this aligns with previous studies indicating that the prevention and treatment of endometriosis remain a formidable challenge in resource-poor countries and regions [9]. Since endometriosis is a complex gynecological condition influenced by the interplay of hormones, inflammation, and angiogenesis, making the elucidation of its specific pathogenic mechanisms and the development of targeted therapies a significant challenge. Current research indicates that signaling pathways such as estrogen receptor [35], MAPK/ERK [36], PI3K/AKT [37], Wnt/β-catenin [38], and angiogenesis-related pathways (e.g. VEGF) play crucial roles in the onset and progression of endometriosis [39]. These pathways interact and collectively promote the growth and spread of lesions. Future research should focus on understanding the mechanisms underlying these signaling pathways and their interactions, which will aid in the development of more precise and effective therapeutic strategies [40]. In the study of uterine fibroids, we found the highest ASIR and ASPR in countries with high SDI, which may be due to more abundant medical resources and comprehensive healthcare systems, leading to increased diagnostic rates. Lou et al. employed an Age-Period-Cohort model to estimate changes in the incidence of uterine fibroids and found a significant burden in regions with middle, lower-middle, and low SDI quintiles [41], but they observed a lower incidence of fibroids in the high SDI group (using net drift), which differs from our study (using EAPC). These findings suggest that these lower SDI regions face both a high disease burden and poorer disease outcomes, advocating for improved screening and treatment of gynecological diseases in women, the widespread implementation of early diagnostic and treatment programs, and the development and promotion of advanced diagnostic and treatment technologies in these areas.

For gynecological cancers, cervical cancer remains a significant health issue among young women, with the effectiveness of its prevention strategies significantly proven by vaccination against the HPV and timely screening and treatment of precancerous lesions [15]. The study found that the higher the SDI quintile, the lower the burden of cervical cancer, with the lowest burden observed in the highest SDI quintiles. This trend is largely attributable to the expansion of HPV vaccination coverage and population-based cervical cancer screening programs. A systematic review and meta-analysis demonstrated a 54–83% reduction in high-risk HPV infections and a 31–51% decrease in precancerous lesions among young women in high-income countries with high vaccination coverage [42]. Moreover, comprehensive cervical cytology screening programs in high-income countries have achieved a 40–90% reduction in the incidence and mortality rates of cervical cancer [43, 44]. Despite this, the proportion of cervical cancer among older women remains high, emphasizing the need for effective vaccination in this population. In contrast, countries and regions with low SDI have the highest burden of cervical cancer, showing the highest age-standardized incidence rates, DALYs, and mortality rates. This is directly linked to poverty, lack of screening and treatment resources, and insufficient infrastructure in these areas [45]. It has been reported that less than 3% of females aged 10 to 20 in developing countries have received the HPV vaccine, compared to over 33.6% in more developed regions [46]. Additionally, only about 20% of women in developing countries have undergone cervical cancer screening, compared to over 60% in more developed regions [47]. This highlights the urgent need to broadly promote HPV vaccination in low-income areas. The overall five-year survival rate for advanced cervical cancer patients is below 19% (source: Centers for Disease Control and Prevention United States cancer statistics). The World Health Assembly’s resolution on “Eliminating Cervical Cancer” passed in 2020 set specific targets for 2030, including 90% of girls being vaccinated with the HPV vaccine before the age of 15, 70% of women aged 35–45 undergoing high-efficacy testing and screening, and 90% of precancerous and invasive cancer cases receiving treatment [48]. In low-income countries, Visual Inspection with Acetic acid as a cost-effective alternative screening tool, despite a higher false-positive rate, is worth considering for application in primary care [49]. In addition, for recurrent cervical cancer, current novel therapies are progressing towards personalized treatment approaches [50–52]. Long non-coding RNA FOXP4-AS1 plays a significant role in the development of cervical cancer by regulating the miR-136-5p/CBX4 axis, thus promoting cancer progression [51, 52]. Furthermore, another study identified five miRNAs, including hsa-miR-136-5p, through the expression screening, which may be closely associated with the circRNA-related ceRNA network in cervical cancer. These miRNAs are potential biomarkers for prognostic evaluation of cervical cancer [53]. For ovarian cancer, the study found that high SDI is associated with an increased burden of ovarian cancer. This may be related to socioeconomic development, a decrease in the number of births, a decline in breastfeeding, and increases in infertility and obesity, which are known risk factors for ovarian cancer. The ASIR and ASPR for ovarian cancer are the lowest in low SDI regions, which may be attributed to differences in the social environment faced by women in different SDI areas. Studies suggest that pregnancy has a protective effect against ovarian cancer, as cessation of ovulation during pregnancy and breastfeeding can reduce damage to the ovarian epithelium. However, in developed countries, women typically have lower fertility rates [54]. Additionally, women in low SDI areas often consume fewer processed foods and engage in more intense physical activity, which may help reduce the incidence of ovarian cancer. Nonetheless, due to lower medical standards, missed diagnoses remain a significant issue in these regions. Further research is needed to develop region-specific prevention and treatment strategies for ovarian cancer, particularly in low-income areas [55]. As for uterine cancer, large epidemiological studies have shown that obesity, hormonal imbalances, and metabolic factors are key risk factors [56]. In regions characterized by high and upper-middle SDI levels, uterine cancer has become an increasingly serious health concern due to factors such as an aging population, declining fertility rates, and rising levels of obesity. These areas possess comprehensive healthcare systems that, through early detection and diagnosis, have increased the reported incidence of uterine cancer. Therefore, uterine cancer has become one of the most common gynecological cancers in high SDI areas [57].

The burden of six gynecological conditions exhibits distinct age patterns. For non-malignant gynecological diseases, the age pattern from the GBD 2019 study previously indicated that the incidence of PCOS peaks among adolescent females aged 15 to 19, a pattern that persisted in 2021 [2]. The primary characteristics of adolescent PCOS patients are menstrual irregularities and hyperandrogenism [58]. Obesity and associated insulin resistance are considered major contributors to PCOS [59]. A significant rise in obesity among girls in recent decades has led to an increased prevalence of adolescent PCOS [58]. The main drivers of adolescent obesity can be attributed to changes in diet and lifestyle, such as sedentary behavior, lack of exercise, consumption of ultra-processed foods and sugary drinks, and circadian rhythm disruptions. Exposure to endocrine-disrupting chemicals, particularly bisphenol A, has also been implicated in the increased incidence of PCOS [60].

The health impacts of PCOS are multifaceted. Reproductively, PCOS can lead to ovulatory issues, menstrual irregularities, and even premature menopause, potentially resulting in infertility. Metabolically, patients with PCOS face significantly increased risks of chronic diseases such as cardiovascular disease and diabetes. Moreover, PCOS is often associated with mood swings, anxiety, depression, and other psychological challenges [61]. Sustained lifestyle interventions have normalizing effects on circulating androgens, insulin resistance, cardiovascular risk factors, and reproductive health outcomes and are considered first-line treatment for adolescent PCOS [58]. Additionally, raising awareness of PCOS symptoms among adolescent females can aid in early detection. However, diagnosing PCOS in adolescent girls may be challenging, as the initial features of PCOS, including polycystic ovarian morphology, menstrual irregularities, and acne, can be normal aspects of pubertal development [62]. Thus, while there are three sets of diagnostic criteria for defining the condition in mature women, their application in adolescents remains controversial [63]. Pediatric and gynecological healthcare providers must use accurate methods to differentiate the early manifestations of PCOS from the physiological characteristics of puberty. For endometriosis, our data indicate that the prevalence varies by age group, with the highest burden among young and middle-aged women aged 20 to 24, decreasing with advancing age. This suggests that the potential late-age burden of infertility related to endometriosis may become a significant issue in the future, necessitating early attention and intervention [9]. Additionally, the potential association between endometriosis and ovarian cancer should not be overlooked. Recent studies suggest that endometriosis may increase the risk of transforming into ovarian cancer [64]. This study has found that the age distribution patterns of endometriosis and ovarian cancer alternate, with the peak of occurrence for endometriosis occurring earlier and ovarian cancer later, which may further suggest a relationship between the two. More research is needed to investigate this association and clarify its mechanisms and risk factors. The situation with uterine fibroids is closely related to age. Our study indicates that the incidence of uterine fibroids begins to significantly increase from ages 10 to 14, peaking at ages 35 to 39, and then gradually decreases with age [41]. Previous studies have shown that the prevalence of uterine fibroids tends to increase with age during the reproductive years and decrease after menopause [65–68], which is consistent with our findings. Indeed, uterine fibroids are highly dependent on ovarian steroid hormones, estrogen, and progesterone [69]. Ovarian activity is crucial for the growth of uterine fibroids, which are not detected in prepubescent girls and most fibroids shrink after menopause [70, 71]. Therefore, screening for uterine fibroids, long-term follow-up, and appropriate treatment for women of reproductive age are necessary measures to alleviate the global health burden.

For gynecological cancers, the incidence, prevalence, and DALYs of cervical cancer remain primarily concentrated in the 40–59 age group. However, studies have identified an increasing incidence of cervical cancer among younger women globally, particularly in regions with a higher SDI [72]. This trend may be linked to increased exposure to HPV, higher participation rates in screening programs, earlier onset of sexual activity, and histories of multiple sexual partners [73]. Additionally, environmental pollution and exposure to endocrine disruptors have also impacted the risk of cervical cancer in younger populations [74]. Given this trend, it is necessary to advance the timing of HPV-based screening programs for high-risk groups to enable early intervention and treatment. Furthermore, age is an independent negative prognostic factor for cervical cancer, with older patients often receiving more conservative treatments [75], while younger patients demonstrate a more proactive attitude towards disease awareness and health behaviors [76]. For ovarian cancer, the overall burden is heaviest among women aged 50 to 69, with mortality rates being most prevalent among those aged 65 to 69. This aligns with the conclusions of previous studies [77, 78] and may relate to population aging. The two main types of epithelial ovarian cancer show that patients under 40 mostly have Type I tumors, which generally present early and have a good prognosis. In older patients, Type II epithelial tumors, which account for about 75%, often present late and have a poor prognosis [79]. Additionally, the elderly have more comorbidities and more adverse biological factors [80], which may also contribute to the differences in incidence and mortality burdens of ovarian cancer across age groups. Currently, there is a clear trend of ovarian cancer occurring in younger women, with approximately 12% of ovarian cancer patients being of reproductive age [81]. Each year, there are about 38,500 new cases of ovarian cancer in women aged 15–40. For young ovarian cancer patients who wish to have children, preserving fertility is just as crucial as focusing on treatment outcomes. A review by Zhang et al. indicates that the decision to perform fertility-sparing surgery primarily depends on factors such as the patient’s age, fertility desires, reproductive plans, tumor histological type, pathological grade, and FIGO stage [82–84]. Regarding uterine cancer, our research finds that its incidence, prevalence, and DALYs are mainly concentrated in postmenopausal women aged 55 to 69, also highlighting the significant impact of population aging on the trends of uterine cancer. Therefore, as the trend of population aging intensifies, prevention, screening, and treatment strategies for uterine cancer need to be optimized for this specific age group of women to mitigate the health burden [85].

In 2021, high BMI, smoking, unsafe sex, and occupational exposure to asbestos were identified as driving factors for the burden of gynecological cancers. Globally, approximately 1.00% of cervical cancer deaths and DALYs could be attributed to unsafe sex, a concerning figure given that cervical cancer is the second most significant disease caused by unsafe sexual practices. Previous research has shown an upward trend in cervical cancer deaths and DALYs resulting from unsafe sex from 1990 to 2019. Encouragingly, the ASDR and ASIR for cervical cancer related to unsafe sex have been declining [86], indicating that effective prevention and control measures for unsafe sexual behaviors contribute to reducing the risk of sexually transmitted diseases. Smoking, as a risk factor for cervical cancer, may increase the risk of high-grade cervical lesions in women with persistent high-risk HPV infections, contributing to approximately 0.08% of deaths and 0.07% of DALYs attributable to smoking [87], underscoring the importance of smoking cessation in cervical cancer prevention. Excess adiposity and high BMI are considered significant contributors to most cancers [88, 89]. Numerous studies have reported an association between increased BMI and the risk of uterine cancer [90–94], and a stronger relationship between BMI and the risk of endometrioid adenocarcinoma [95, 96]. Current genome-wide association studies have identified high BMI as one of the clearest risk factors for uterine cancer, identifying risk loci and the basis for risk stratification [97]. Obesity is associated not only with high levels of circulating estrogens, abnormal fatty acid metabolism, and chronic inflammation [98] but may also affect the mapping of sentinel lymph nodes during minimally invasive surgery [99–102], providing new intervention points for treatment. Therefore, understanding the significant burden of uterine cancer associated with high BMI is essential for effectively addressing both individual and societal health outcomes. Providing timely health monitoring and disease control for populations at high risk of obesity-related cancers is critical to reducing the overall burden. In light of this, integrating clinical management strategies for obesity into the treatment of uterine cancers is necessary to mitigate the associated mortality risks [103]. Moreover, our research indicates that a high BMI is a specific risk factor for ovarian cancer mortality in 2021. The current lifestyle and the recent increase in obesity have played roles in increasing the incidence of ovarian cancer [104, 105] Theoretically, red meat is associated with an increased risk of ovarian cancer due to its content of various mutagens, such as carcinogenic heterocyclic amines, endogenous formation of nitroso compounds, fats, salt, and iron [106]. Therefore, many studies now suggest a correlation between obesity and an increased risk of ovarian cancer, as well as an association with reduced survival rates specific to ovarian cancer. In the Women’s Health Initiative cohort study, a low-fat dietary pattern and physical activity showed a potential inverse correlation with ovarian cancer risk [107], offering effective solutions for individuals to avoid the risk of ovarian cancer. Occupational exposure to asbestos is another specific risk factor for ovarian cancer mortality, accounting for about 0.03% of all deaths. Current research consistently shows that human mesothelial cells are highly sensitive to asbestos toxicity [108], but further scientific research is urgently needed to clarify the causal relationship between asbestos and ovarian cancer. Despite the ban on asbestos in many countries [109], exposure risks still exist in certain regions. The causality between asbestos exposure and ovarian cancer requires more scientific elucidation. Considering the ongoing process of industrialization, appropriate policies should not only limit the exposure to asbestos but prohibit its use or manage structures already containing asbestos to reduce cancer mortality [110].

### Limitation

This study, based on the updated estimates from the GBD 2021, presents the global burden of six common gynecological diseases, yet there are several limitations to consider. Firstly, the estimation of the burden of gynecological diseases relies heavily on the availability and quality of GBD 2021 data. In certain countries/regions, particularly in low- and middle-income areas, access to original/raw data may be limited, which could hinder the estimations by GBD researchers. Secondly, the COVID-19 pandemic has introduced significant uncertainty into mortality estimates for all diseases, especially in regions most heavily affected by the pandemic. Thirdly, a narrow focus on statistical significance testing may overlook the clinical relevance of study findings. To mitigate this limitation, we advocate for the development and implementation of different analytical approaches to broaden and validate the results of this study. Fourthly, due to the limitations in the definitions of gynecological diseases provided by the GBD, the burden of disease may be underestimated. Lastly, the GBD’s risk factor analysis, based on literature review, may not encompass all risk factors for each disease.

## Conclusions

The GBD data from 2021 elucidate the complexity and diversity of the burden of non-malignant gynecological diseases and gynecological cancers on a global scale. Uterine fibroids, as the predominant non-malignant gynecological condition, have shown an increasing trend in incidence and prevalence across all age groups, underscoring the urgent need for effective prevention and treatment strategies. Cervical cancer contributes substantially to the mortality and morbidity of malignant gynecological diseases, indicating the necessity to strengthen early screening and preventive measures, particularly in regions with low to medium development indices. Long-term trends reveal the growing importance of preventive measures and lifestyle interventions, as evidenced by the rising prevalence of PCOS and endometriosis, alongside the declining incidence rates of cervical and ovarian cancers. Furthermore, the significant disparities in the burden of gynecological diseases across regions with different socio-developmental indices call for more precise public health policies and resource allocation tailored to specific populations and areas. Lastly, unsafe sexual behaviors and high body mass index as attributable risk factors for diseases such as cervical and ovarian cancers highlight the necessity of health education and the promotion of healthy lifestyles. These findings provide a critical foundation for formulating effective global and regional health strategies to alleviate the global burden of gynecological diseases.

## Electronic supplementary material

Below is the link to the electronic supplementary material.

Supplementary Material 1

## Data Availability

The data underlying this article will be provided by the corresponding author upon reasonable request.
