# Supplementary material for: The global burden of polycystic ovary syndrome, endometriosis, uterine fibroids, cervical cancer, uterine cancer, and ovarian cancer from 1990 to 2021
Source: BMC Public Health. 2025 May 14;25:1774. doi: 10.1186/s12889-025-22881-3 (PMC12077057; doi:10.1186/s12889-025-22881-3)
Supplement: Supplementary file 1 — Supplementary Material 1 [file 12889_2025_22881_MOESM1_ESM.docx]

**The Global Burden of Polycystic Ovary Syndrome, Endometriosis, Uterine Fibroids, Cervical Cancer, Uterine Cancer, and Ovarian Cancer from 1990 to 2021**

Wei-Zhen Tang ^a, c^, Qin-Yu Cai ^a, c^, Kang-Jin Huang ^a^，Wei-Ze Xu ^a, c^, Jia-Zheng Li ^a, c^, Yun-Ren Pan ^a, c^, Hong-Yu Xu ^a, c^, Yi-Fan Zhao ^a^, Ting-He Sheng ^b^, Zhi-Mou Li ^a^, Tai-Hang Liu ^a, c,^*,Ying-Bo Li ^a, b,^*

**Affiliations:**

^a^ Department of Bioinformatics, School of Basic Medical Sciences, Chongqing Medical University, Chongqing, 400016, China.

^b^ Institute of Neuroscience, Department of Physiology, School of Basic Medical Science, Chongqing Medical University, Chongqing, 400016, China.

^c^ The Joint International Research Laboratory of Reproduction and Development, Chongqing Medical University, Chongqing, 400016, China.

***Correspondence:**

*Tai-Hang Liu (liuth@cqmu.edu.cn); * Ying-Bo Li (liyingbo@cqmu.edu.cn)

Box 197, Chongqing Medical University, No.1 Yixueyuan Rd, Chongqing, 400016, PR China. Tel.: +86 023 68485868.

Wei-Zhen Tang, [**ORCID**](https://orcid.org/): 0009-0002-8130-7516

Tai-Hang Liu, [**ORCID**](https://orcid.org/): 0000-0001-9932-8281

**Table S1.** List of abbreviations

| Abbreviation | Full Name |
| --- | --- |
| PCOS | Polycystic Ovary Syndrome |
| DALY | Disability-Adjusted Life Year |
| GBD | Global Burden of Disease |
| SDI | Socio-demographic Index |
| EAPC | The Estimated Annual Percentage Change |
| CI | Confidence Interval |
| ASIR | Age-Standardized Incidence Rate |
| ASPR | Age-Standardized Prevalence Rate |
| ASMR | Age-Standardized Mortality Rate |
| ASDR | Age-Standardized Disability Rate |
| BMI | Body Mass Index |
| HPV | Human Papillomavirus |
| COVID-19 | Corona Virus Disease 2019 |
| DW | Disability Weights |
| YLD | Years Lived with Disability |
| YLL | Years of Life Lost |
| TMREL | Theoretical Minimum Risk Exposure Level |
| PAF | Population Attributable Fraction |
| ASR | Age-standardized Rate |
| UI | Uncertainty Interva |
| MAPK | Mitogen-activated Protein Kinase |
| ERK | Extracellular Signal-regulated Kinase |
| PI3K | Phosphoinositide 3-kinase (PI3K) |
| AKT | Protein Kinase B |
| VEGF | Vascular Endothelial Growth Factor |
| miRNA | MicroRNA |
| circRNA | Circular RNA |
| ceRNA | Competing endogenous RNA |
| FIGO | Federation of International of Gynecologists and Obstetricians |

**Table S2.** EAPC of the ASIR, ASPR, ASMR, and ASDR for three non-malignant gynecological diseases globally and across 21 regions.

| **Location** | **EAPC of ASIR (95% CI)** |  |  | **EAPC of ASPR (95% CI)** |  |  | **EAPC of ASMR (95% CI)** |  |  | **EAPC of ASDR (95% CI)** |  |  |
| --- | --- | --- | --- | --- | --- | --- | --- | --- | --- | --- | --- | --- |
|  | **PCOS** | **Endometriosis** | **Uterine fibroids** | **PCOS** | **Endometriosis** | **Uterine fibroids** | **PCOS** | **Endometriosis** | **Uterine fibroids** | **PCOS** | **Endometriosis** | **Uterine fibroids** |
| **Global** | 0.01(-0.10,0.11) | -1.16(-1.21,-1.12) | 0.37(0.30,0.44) | 0.73(0.66,0.80) | -0.92(-0.96,-0.88) | 0.67(0.61,0.73) | 0 | 1.84(1.63,2.05) | 1.04(0.90,1.17) | 0.70(0.62,0.77) | -0.92(-0.96,-0.87) | 0.69(0.61,0.78) |
| **High SDI** | -0.80(-1.01,-0.59) | -1.44(-1.49,-1.39) | -0.34(-0.51,-0.18) | -0.34(-0.51,-0.18) | -1.18(-1.25,-1.10) | 0.04(-0.13,0.21) | 0 | 0.05(-0.17,0.26) | -0.68(-1.06,-0.30) | -0.37(-0.53,-0.20) | -1.18(-1.25,-1.11) | -0.42(-0.49,-0.36) |
| **High-middle SDI** | -0.24(-0.42,-0.07) | -1.17(-1.27,-1.07) | -0.27(-0.37,-0.17) | 0.93(0.75,1.11) | -0.72(-0.79,-0.64) | 0.23(0.13,0.33) | 0 | 0.60(0.32,0.88) | 0.80(0.57,1.04) | 0.90(0.71,1.09) | -0.72(-0.80,-0.65) | 0.09(-0.05,0.24) |
| **Middle SDI** | 0.27(0.12,0.42) | -1.25(-1.32,-1.19) | 0.82(0.70,0.95) | 1.66(1.51,1.81) | -0.82(-0.89,-0.76) | 1.46(1.37,1.55) | 0 | 3.67(3.24,4.10) | 2.67(2.33,3.02) | 1.62(1.47,1.77) | -0.83(-0.89,-0.76) | 1.70(1.52,1.87) |
| **Low-middle SDI** | 1.09(1.04,1.14) | -1.26(-1.29,-1.24) | 1.18(1.13,1.23) | 1.96(1.92,2.01) | -1.21(-1.24,-1.18) | 1.46(1.39,1.53) | 0 | 2.39(2.10,2.69) | 0.20(0.15,0.25) | 1.90(1.86,1.95) | -1.19(-1.22,-1.16) | 0.46(0.41,0.52) |
| **Low SDI** | 1.31(1.28,1.33) | -1.18(-1.23,-1.12) | 0.57(0.53,0.61) | 1.49(1.45,1.53) | -1.13(-1.20,-1.07) | 0.59(0.52,0.66) | 0 | 1.12(1.07,1.17) | -0.12(-0.27,0.04) | 1.46(1.43,1.49) | -1.10(-1.17,-1.04) | 0.01(-0.08,0.11) |
| **Andean Latin America** | -0.15(-0.33,0.03) | -1.09(-1.14,-1.04) | 0.58(0.55,0.60) | 1.28(1.18,1.38) | -0.70(-0.77,-0.63) | 1.07(1.04,1.09) | 0 | -2.63(-3.45,-1.80) | -1.48(-1.97,-1.00) | 1.25(1.15,1.35) | -0.70(-0.76,-0.63) | -0.80(-1.09,-0.51) |
| **Australasia** | -0.55(-0.68,-0.43) | -0.82(-0.92,-0.72) | -0.42(-0.48,-0.35) | -0.16(-0.26,-0.05) | -0.67(-0.75,-0.59) | 0.13(0.03,0.23) | 0 | -3.01(-6.44,0.56) | -5.20(-8.20,-2.11) | -0.16(-0.25,-0.06) | -0.67(-0.75,-0.58) | -1.36(-1.88,-0.84) |
| **Caribbean** | 0.04(-0.10,0.18) | -1.06(-1.13,-1.00) | 0.05(-0.08,0.17) | 0.68(0.58,0.78) | -0.82(-0.86,-0.77) | 0.74(0.64,0.84) | 0 | 0.24(-0.86,1.36) | -0.51(-0.70,-0.32) | 0.65(0.55,0.75) | -0.82(-0.86,-0.78) | -0.37(-0.53,-0.22) |
| **Central Asia** | 0.16(-0.09,0.41) | -0.22(-0.43,-0.01) | 0.69(0.56,0.82) | 1.47(1.35,1.59) | 0.04(-0.15,0.24) | 1.22(1.12,1.32) | 0 | 0.48(0.01,0.95) | 1.32(1.16,1.47) | 1.44(1.31,1.56) | 0.05(-0.15,0.25) | 1.14(1.03,1.24) |
| **Central Europe** | -1.21(-1.41,-1.01) | -0.84(-0.85,-0.83) | -0.17(-0.28,-0.06) | 0.22(0.08,0.36) | -0.42(-0.47,-0.37) | 0.17(0.01,0.34) | 0 | -2.87(-4.63,-1.07) | -4.84(-5.67,-4.00) | 0.20(0.06,0.34) | -0.42(-0.47,-0.37) | -1.61(-1.83,-1.39) |
| **Central Latin America** | -1.18(-1.30,-1.06) | -1.33(-1.38,-1.27) | 0.45(0.37,0.52) | 0.01(-0.14,0.16) | -0.93(-0.99,-0.87) | 1.16(1.13,1.20) | 0 | -2.42(-4.20,-0.60) | -0.27(-0.87,0.33) | -0.02(-0.16,0.13) | -0.94(-1.00,-0.88) | -0.02(-0.37,0.33) |
| **Central Sub-Saharan Africa** | 1.36(1.18,1.54) | -1.28(-1.40,-1.15) | 0.34(0.31,0.38) | 1.47(1.31,1.63) | -1.26(-1.39,-1.13) | 0.37(0.34,0.41) | 0 | 2.08(1.91,2.25) | 0.25(0.19,0.32) | 1.46(1.29,1.63) | -1.22(-1.35,-1.09) | 0.04(-0.01,0.09) |
| **East Asia** | -0.26(-0.73,0.21) | -2.02(-2.18,-1.86) | -0.06(-0.37,0.26) | 1.61(1.28,1.95) | -1.45(-1.62,-1.29) | 0.94(0.65,1.23) | 0 | 16.57(13.83,19.38) | 7.15(6.07,8.24) | 1.60(1.26,1.94) | -1.45(-1.62,-1.28) | 3.73(3.11,4.35) |
| **Eastern Europe** | -0.48(-0.75,-0.21) | -0.09(-0.26,0.07) | 0.01(-0.05,0.07) | 0.77(0.63,0.90) | 0.28(0.11,0.45) | 0.40(0.34,0.46) | 0 | -0.31(-0.83,0.22) | -1.35(-1.69,-1.00) | 0.74(0.60,0.88) | 0.28(0.11,0.45) | -0.55(-0.79,-0.32) |
| **Eastern Sub-Saharan Africa** | 0.92(0.88,0.95) | -1.33(-1.39,-1.28) | 0.51(0.46,0.56) | 1.16(1.10,1.22) | -1.18(-1.22,-1.15) | 0.51(0.43,0.60) | 0 | 1.43(1.34,1.52) | -0.66(-0.74,-0.59) | 1.14(1.09,1.19) | -1.15(-1.18,-1.11) | -0.24(-0.30,-0.19) |
| **High-income Asia Pacific** | -1.42(-1.57,-1.28) | -1.43(-1.56,-1.30) | -0.75(-1.00,-0.49) | -0.53(-0.59,-0.47) | -1.19(-1.32,-1.07) | -0.28(-0.40,-0.16) | 0 | 6.76(5.38,8.16) | 1.17(0.79,1.55) | -0.55(-0.61,-0.49) | -1.20(-1.32,-1.07) | -0.31(-0.41,-0.21) |
| **High-income North America** | -0.85(-1.37,-0.32) | -2.71(-2.91,-2.50) | 0.18(-0.13,0.50) | -1.04(-1.56,-0.52) | -2.46(-2.66,-2.26) | 0.39(0.10,0.68) | 0 | 0.14(-0.15,0.42) | 0.32(0.16,0.48) | -1.06(-1.57,-0.55) | -2.47(-2.67,-2.27) | 0.37(0.17,0.58) |
| **North Africa and Middle East** | -0.09(-0.25,0.06) | -1.06(-1.10,-1.03) | 0.81(0.73,0.89) | 1.45(1.29,1.61) | -0.70(-0.74,-0.65) | 1.14(1.09,1.19) | 0 | 2.83(2.71,2.95) | -0.24(-0.30,-0.18) | 1.37(1.20,1.54) | -0.70(-0.75,-0.65) | 0.19(0.14,0.24) |
| **Oceania** | 0.45(0.24,0.66) | -0.50(-0.54,-0.45) | 0.51(0.43,0.58) | 1.00(0.80,1.20) | -0.28(-0.33,-0.24) | 0.75(0.71,0.80) | 0 | 3.24(2.95,3.52) | 0.29(0.24,0.35) | 0.98(0.78,1.18) | -0.28(-0.32,-0.23) | 0.27(0.22,0.31) |
| **South Asia** | 1.50(1.40,1.59) | -1.27(-1.30,-1.25) | 1.43(1.33,1.53) | 2.52(2.40,2.64) | -1.26(-1.29,-1.22) | 1.73(1.60,1.86) | 0 | 1.34(1.19,1.50) | -0.28(-0.35,-0.20) | 2.45(2.34,2.56) | -1.24(-1.27,-1.20) | 0.30(0.23,0.36) |
| **Southeast Asia** | 1.25(1.13,1.38) | -0.86(-0.92,-0.79) | 0.42(0.31,0.54) | 2.38(2.22,2.54) | -0.50(-0.57,-0.43) | 1.08(0.98,1.17) | 0 | 4.67(4.27,5.07) | 0.29(0.22,0.36) | 2.32(2.17,2.48) | -0.49(-0.55,-0.42) | 0.54(0.48,0.60) |
| **Southern Latin America** | 0.69(0.37,1.00) | -0.39(-0.48,-0.31) | 0.38(0.27,0.49) | 1.57(1.33,1.82) | -0.15(-0.27,-0.03) | 0.62(0.50,0.75) | 0 | 4.92(3.65,6.21) | -3.34(-4.07,-2.61) | 1.56(1.32,1.80) | -0.15(-0.27,-0.03) | -1.30(-1.60,-1.00) |
| **Southern Sub-Saharan Africa** | -0.12(-0.32,0.08) | -0.70(-0.78,-0.62) | 0.58(0.54,0.62) | 1.01(0.87,1.15) | -0.34(-0.38,-0.31) | 0.89(0.84,0.94) | 0 | 5.81(3.80,7.85) | 1.98(1.61,2.34) | 0.96(0.82,1.10) | -0.37(-0.40,-0.33) | 1.36(1.11,1.61) |
| **Tropical Latin America** | -1.66(-1.85,-1.47) | -1.38(-1.52,-1.24) | 1.79(1.68,1.90) | -0.10(-0.25,0.06) | -0.83(-1.02,-0.64) | 2.06(1.97,2.15) | 0 | 3.76(3.35,4.17) | 3.20(3.01,3.39) | -0.14(-0.29,0.01) | -0.81(-1.01,-0.62) | 2.33(2.22,2.45) |
| **Western Europe** | -0.54(-0.63,-0.46) | -0.62(-0.72,-0.52) | -0.42(-0.56,-0.29) | -0.25(-0.35,-0.15) | -0.54(-0.60,-0.47) | 0.16(0.04,0.28) | 0 | -3.02(-3.58,-2.45) | -2.92(-3.40,-2.43) | -0.27(-0.37,-0.17) | -0.54(-0.61,-0.48) | -0.94(-1.04,-0.84) |
| **Western Sub-Saharan Africa** | 0.87(0.71,1.04) | -1.12(-1.22,-1.01) | 0.29(0.20,0.38) | 1.07(0.84,1.30) | -0.90(-1.00,-0.79) | 0.41(0.36,0.47) | 0 | 2.07(1.55,2.59) | 0.86(0.41,1.31) | 1.06(0.83,1.29) | -0.87(-0.98,-0.77) | 0.67(0.33,1.01) |

**Table S3.** EAPC of the ASIR, ASPR, ASMR, and ASDR for three gynecological cancers globally and across 21 regions.

| **Location** | **EAPC of ASIR (95% CI)** |  |  | **EAPC of ASPR (95% CI)** |  |  | **EAPC of ASMR (95% CI)** |  |  | **EAPC of ASDR (95% CI)** |  |  |
| --- | --- | --- | --- | --- | --- | --- | --- | --- | --- | --- | --- | --- |
|  | **Cervical cancer** | **Ovarian cancer** | **Uterine cancer** | **Cervical cancer** | **Ovarian cancer** | **Uterine cancer** | **Cervical cancer** | **Ovarian cancer** | **Uterine cancer** | **Cervical cancer** | **Ovarian cancer** | **Uterine cancer** |
| **Global** | 0.33(0.24,0.42) | 0.66(0.62,0.70) | 1.74(1.65,1.83) | 0.80(0.74,0.87) | 0.80(0.74,0.86) | 1.93(1.84,2.01) | -0.20(-0.31,-0.10) | 0.60(0.56,0.64) | 0.50(0.38,0.62) | -0.36(-0.45,-0.27) | 0.48(0.44,0.52) | -0.78(-0.85,-0.71) |
| **High SDI** | -0.97(-1.11,-0.83) | -0.25(-0.37,-0.14) | 2.30(2.19,2.42) | -0.79(-0.92,-0.67) | -0.21(-0.38,-0.04) | 2.48(2.38,2.59) | -1.09(-1.21,-0.97) | -0.13(-0.20,-0.05) | 1.24(1.03,1.45) | -1.35(-1.45,-1.26) | -0.50(-0.58,-0.41) | 0.26(0.11,0.42) |
| **High-middle SDI** | 1.21(1.11,1.31) | 0.70(0.63,0.77) | 1.97(1.83,2.11) | 1.97(1.82,2.11) | 0.81(0.72,0.90) | 2.16(2.01,2.31) | 0.12(0.06,0.18) | 0.76(0.70,0.82) | 0.34(0.21,0.47) | -0.00(-0.06,0.06) | 0.43(0.37,0.49) | -1.27(-1.38,-1.15) |
| **Middle SDI** | 1.07(1.01,1.12) | 2.26(2.21,2.32) | 2.47(2.39,2.55) | 1.74(1.70,1.79) | 2.42(2.36,2.47) | 2.79(2.71,2.87) | 0.32(0.24,0.40) | 2.25(2.17,2.34) | 0.76(0.66,0.86) | 0.04(-0.03,0.11) | 1.97(1.90,2.05) | -1.29(-1.40,-1.18) |
| **Low-middle SDI** | 0.23(0.05,0.40) | 2.58(2.53,2.62) | 2.24(2.08,2.40) | 0.74(0.58,0.90) | 2.76(2.71,2.81) | 2.47(2.29,2.65) | -0.29(-0.48,-0.11) | 2.56(2.51,2.61) | 1.37(1.26,1.47) | -0.51(-0.68,-0.34) | 2.37(2.32,2.41) | 0.04(-0.03,0.11) |
| **Low SDI** | -0.95(-1.13,-0.77) | 1.40(1.23,1.58) | 0.62(0.41,0.83) | -0.33(-0.52,-0.13) | 1.80(1.63,1.98) | 0.93(0.69,1.16) | -1.41(-1.58,-1.25) | 1.17(1.00,1.35) | -0.02(-0.18,0.13) | -1.48(-1.64,-1.32) | 1.15(0.97,1.33) | -0.19(-0.27,-0.10) |
| **Andean Latin America** | 0.79(0.63,0.95) | 3.58(3.15,4.00) | 2.17(1.96,2.37) | 1.67(1.51,1.84) | 3.86(3.38,4.35) | 2.51(2.29,2.73) | 0.06(-0.08,0.20) | 3.55(3.19,3.92) | 0.79(0.58,0.99) | -0.31(-0.47,-0.16) | 3.29(2.90,3.67) | -1.13(-1.34,-0.93) |
| **Australasia** | -1.52(-1.75,-1.29) | -1.22(-1.62,-0.83) | 2.46(2.26,2.65) | -1.15(-1.39,-0.90) | -1.27(-1.72,-0.82) | 2.72(2.54,2.90) | -2.29(-2.50,-2.09) | -1.01(-1.38,-0.64) | 1.48(1.21,1.75) | -2.55(-2.78,-2.31) | -1.47(-1.83,-1.12) | 0.32(0.07,0.56) |
| **Caribbean** | 0.51(0.42,0.61) | 2.09(1.90,2.28) | 2.49(2.23,2.76) | 0.73(0.57,0.89) | 2.08(1.84,2.32) | 2.49(2.20,2.78) | 0.36(0.30,0.43) | 2.25(2.10,2.39) | 2.25(2.09,2.42) | 0.19(0.13,0.25) | 2.05(1.89,2.21) | 0.64(0.45,0.83) |
| **Central Asia** | 0.47(0.29,0.65) | 2.09(1.91,2.26) | 0.70(0.31,1.09) | 0.86(0.67,1.06) | 2.22(2.04,2.41) | 0.93(0.54,1.33) | -0.18(-0.33,-0.03) | 2.04(1.87,2.21) | -0.40(-0.74,-0.05) | -0.08(-0.24,0.09) | 2.01(1.84,2.17) | -1.14(-1.40,-0.88) |
| **Central Europe** | -0.29(-0.51,-0.08) | 1.22(1.04,1.40) | 2.63(2.53,2.73) | -0.08(-0.36,0.20) | 0.97(0.75,1.20) | 2.77(2.68,2.85) | -0.57(-0.71,-0.43) | 1.62(1.46,1.78) | 1.62(1.40,1.84) | -1.11(-1.30,-0.92) | 0.98(0.81,1.15) | -0.19(-0.36,-0.02) |
| **Central Latin America** | 0.11(-0.06,0.29) | 3.13(3.06,3.20) | 3.83(3.41,4.25) | 0.74(0.58,0.90) | 3.20(3.11,3.29) | 4.23(3.82,4.64) | -0.69(-0.84,-0.55) | 3.27(3.21,3.33) | 2.61(2.18,3.04) | -0.80(-0.95,-0.64) | 3.04(2.99,3.10) | 0.38(-0.01,0.78) |
| **Central Sub-Saharan Africa** | -0.06(-0.14,0.03) | 1.57(1.29,1.86) | 0.69(0.44,0.93) | 0.58(0.41,0.75) | 1.98(1.65,2.30) | 1.00(0.70,1.31) | -0.49(-0.53,-0.45) | 1.34(1.08,1.61) | 0.05(-0.09,0.18) | -0.48(-0.52,-0.43) | 1.40(1.12,1.69) | 0.17(0.10,0.24) |
| **East Asia** | 2.48(2.28,2.67) | 1.49(1.38,1.60) | 2.71(2.36,3.06) | 3.57(3.31,3.84) | 1.78(1.72,1.85) | 3.13(2.73,3.53) | 1.08(0.91,1.24) | 1.41(1.24,1.58) | -0.13(-0.44,0.17) | 0.72(0.56,0.88) | 1.07(0.93,1.21) | -2.39(-2.72,-2.06) |
| **Eastern Europe** | 0.46(0.35,0.57) | 0.52(0.33,0.71) | 1.69(1.22,2.17) | 1.44(1.30,1.59) | 0.47(0.29,0.66) | 1.81(1.31,2.31) | -0.79(-0.88,-0.70) | 0.64(0.46,0.82) | 0.49(0.07,0.91) | -0.43(-0.54,-0.32) | 0.30(0.11,0.49) | -0.48(-0.88,-0.07) |
| **Eastern Sub-Saharan Africa** | -1.02(-1.22,-0.82) | 1.41(1.21,1.61) | 0.52(0.28,0.77) | -0.35(-0.57,-0.12) | 1.86(1.65,2.06) | 0.90(0.63,1.16) | -1.50(-1.69,-1.32) | 1.16(0.95,1.37) | -0.12(-0.32,0.07) | -1.55(-1.74,-1.37) | 1.18(0.98,1.38) | -0.36(-0.44,-0.27) |
| **High-income Asia Pacific** | 0.64(0.56,0.73) | 1.32(1.14,1.50) | 3.48(3.31,3.65) | 1.06(0.92,1.20) | 1.43(1.16,1.70) | 3.71(3.49,3.93) | -0.04(-0.11,0.03) | 1.45(1.35,1.55) | 2.05(1.92,2.18) | -0.46(-0.52,-0.40) | 0.55(0.46,0.65) | 0.30(0.12,0.49) |
| **High-income North America** | -1.08(-1.29,-0.87) | -0.45(-0.60,-0.30) | 2.65(2.48,2.82) | -1.17(-1.38,-0.97) | -0.54(-0.73,-0.36) | 2.83(2.66,2.99) | -0.26(-0.38,-0.14) | -0.27(-0.41,-0.14) | 1.60(1.33,1.87) | -0.53(-0.64,-0.42) | -0.49(-0.62,-0.37) | 1.00(0.85,1.16) |
| **North Africa and Middle East** | 0.49(0.42,0.56) | 2.43(2.37,2.49) | 3.36(3.10,3.62) | 1.02(0.96,1.08) | 2.71(2.65,2.77) | 3.59(3.33,3.85) | -0.22(-0.31,-0.13) | 2.34(2.25,2.43) | 1.31(1.04,1.57) | -0.43(-0.50,-0.36) | 2.14(2.06,2.21) | 0.02(-0.17,0.22) |
| **Oceania** | -0.19(-0.28,-0.10) | 1.80(1.70,1.89) | 1.20(1.04,1.36) | -0.13(-0.24,-0.03) | 1.98(1.85,2.11) | 1.29(1.11,1.47) | -0.18(-0.28,-0.09) | 1.65(1.61,1.70) | 1.03(0.91,1.15) | -0.22(-0.30,-0.14) | 1.68(1.63,1.72) | 0.44(0.40,0.48) |
| **South Asia** | -0.37(-0.73,0.00) | 2.62(2.50,2.73) | 2.53(2.25,2.80) | 0.19(-0.16,0.54) | 2.81(2.70,2.92) | 2.81(2.52,3.11) | -0.89(-1.26,-0.53) | 2.59(2.47,2.70) | 1.50(1.31,1.69) | -1.17(-1.52,-0.83) | 2.33(2.20,2.45) | -0.11(-0.27,0.05) |
| **Southeast Asia** | 0.62(0.51,0.73) | 2.69(2.58,2.80) | 2.70(2.62,2.79) | 0.95(0.81,1.10) | 2.85(2.69,3.01) | 2.87(2.78,2.97) | 0.24(0.16,0.32) | 2.71(2.68,2.74) | 1.73(1.67,1.80) | -0.07(-0.17,0.02) | 2.49(2.44,2.55) | -0.05(-0.14,0.04) |
| **Southern Latin America** | 0.39(0.23,0.55) | 0.54(0.37,0.70) | 0.83(0.68,0.98) | 0.87(0.70,1.05) | 0.81(0.63,1.00) | 1.02(0.86,1.18) | -0.23(-0.37,-0.09) | 0.45(0.29,0.61) | -0.19(-0.31,-0.06) | -0.41(-0.55,-0.27) | 0.28(0.15,0.42) | -1.31(-1.46,-1.16) |
| **Southern Sub-Saharan Africa** | 2.92(2.36,3.48) | 2.60(2.46,2.74) | 3.59(3.36,3.82) | 2.85(2.25,3.44) | 2.39(2.25,2.53) | 3.67(3.48,3.87) | 2.83(2.33,3.33) | 2.78(2.67,2.89) | 3.05(2.75,3.35) | 2.76(2.17,3.34) | 2.65(2.52,2.79) | 1.90(1.55,2.24) |
| **Tropical Latin America** | 0.66(0.51,0.82) | 1.84(1.75,1.93) | 2.46(2.27,2.66) | 1.21(1.02,1.40) | 1.83(1.74,1.93) | 2.71(2.49,2.92) | 0.10(-0.01,0.21) | 2.04(1.96,2.13) | 1.57(1.40,1.73) | -0.09(-0.22,0.04) | 1.68(1.60,1.76) | -0.66(-0.81,-0.51) |
| **Western Europe** | -0.86(-1.05,-0.67) | -0.52(-0.60,-0.44) | 2.14(1.98,2.30) | -0.57(-0.72,-0.42) | -0.51(-0.66,-0.37) | 2.28(2.09,2.46) | -1.16(-1.39,-0.93) | -0.29(-0.34,-0.24) | 1.18(1.03,1.33) | -1.46(-1.66,-1.26) | -0.78(-0.83,-0.72) | 0.23(0.08,0.39) |
| **Western Sub-Saharan Africa** | -0.12(-0.17,-0.08) | 1.65(1.48,1.82) | 1.06(0.91,1.20) | 0.32(0.26,0.37) | 2.04(1.88,2.19) | 1.41(1.25,1.58) | -0.58(-0.62,-0.54) | 1.34(1.15,1.53) | 0.34(0.23,0.44) | -0.58(-0.62,-0.53) | 1.53(1.35,1.70) | 0.62(0.58,0.67) |

**Table S4.** Regional prevalence and ASPR of six gynecological diseases in 2021.

| **Location** | **PCOS** |  | **Endometriosis** |  | **Uterine fibroids** |  | **Cervical cancer** |  | **Ovarian cancer** |  | **Uterine cancer** |  |
| --- | --- | --- | --- | --- | --- | --- | --- | --- | --- | --- | --- | --- |
|  | **Prevalence (× 105, 95% UI)** | **ASPR (1/100,000, 95% UI)** | **Prevalence (× 105, 95% UI)** | **ASPR (1/100,000, 95% UI)** | **Prevalence (× 105, 95% UI)** | **ASPR (1/100,000, 95% UI)** | **Prevalence (× 105, 95% UI)** | **ASPR (1/100,000, 95% UI)** | **Prevalence (× 105, 95% UI)** | **ASPR (1/100,000, 95% UI)** | **Prevalence (× 105, 95% UI)** | **ASPR (1/100,000, 95% UI)** |
| **Global** | 694.73(495.31,957.24) | 1757.83(1253.36,2421.26) | 222.75(155.17,304.14) | 556.98(388.85,764.05) | 1195.45(912.28,1549.44) | 2841.07(2164.43,3682.27) | 33.85(31.08,36.97) | 79.30(72.81,86.58) | 12.22(11.02,13.32) | 28.08(25.26,30.64) | 34.52(31.65,37.25) | 75.73(69.37,81.78) |
| **High SDI** | 175.74(129.81],238.77) | 3554.29(2624.21,4816.07) | 24.95(18.33,32.62) | 475.97(342.41,633.32) | 205.70(158.94,261.47) | 3184.07(2450.98,4074.76) | 4.68(4.51,4.83) | 68.67(66.45,70.84) | 3.16(2.96,3.30) | 37.89(35.88,39.41) | 14.48(13.46,15.06) | 152.48(143.48,158.54) |
| **High-middle SDI** | 111.81(78.75,155.91) | 1817.62(1277.35,2529.47) | 34.11(24.38,45.32) | 513.77(363.98,689.66) | 237.15(181.50,306.02) | 2913.18(2224.94,3741.19) | 6.78(5.81,7.80) | 79.72(68.44,91.34) | 2.76(2.42,3.09) | 30.37(26.64,34.23) | 10.98(9.98,12.07) | 106.73(96.90,117.72) |
| **Middle SDI** | 246.13(174.53,340.74) | 1971.07(1395.79,2724.23) | 65.49(46.02,89.77) | 506.23(354.30,696.05) | 363.01(277.08,467.26) | 2600.71(1987.89,3347.17) | 11.95(10.63,13.38) | 83.80(74.69,93.87) | 3.57(3.06,4.00) | 25.43(21.73,28.62) | 6.17(4.98,7.28) | 41.43(33.43,48.85) |
| **Low-middle SDI** | 121.18(84.34,170.27) | 1188.74(828.69,1668.94) | 60.92(42.18,84.92) | 603.63(417.71,842.37) | 275.47(206.90,360.78) | 2600.71(1987.89,3347.17) | 6.82(6.07,7.59) | 74.93(66.83,83.29) | 2.04(1.75,2.40) | 22.92(19.71,26.94) | 2.20(1.89,2.68) | 26.88(23.09,32.67) |
| **Low SDI** | 39.38(27.45,55.90) | 714.31(504.52,1007.40) | 37.10(25.12,51.80) | 714.86(485.20,987.94) | 113.14(85.40,148.58) | 2703.75(2040.97,3559.58) | 3.59(3.00,4.30) | 90.16(75.64,107.64) | 0.69(0.51,0.83) | 18.42(13.85,22.07) | 0.64(0.51,0.82) | 21.28(17.03,27.43) |
| **Andean Latin America** | 11.73(808337.39,1649165.85) | 3333.66(2301.61,4690.91) | 1.55(1.07,216870.53) | 435.83(299.30,606.35) | 20.89(15.94,27.11) | 6101.66(4649.90,7894.76) | 0.57(0.43,0.72) | 170.17(129.85,216.09) | 0.09(0.07,0.12) | 28.51(21.49,36.37) | 0.21(0.16,0.28) | 67.19(49.68,87.00) |
| **Australasia** | 7.02(5.00,9.75) | 4786.95(3430.23,6607.27) | 0.81(0.57,1.11) | 539.47(380.69,738.21) | 1.79(1.36,2.34) | 979.30(737.69,1282.38) | 0.12(0.11,0.13) | 60.80(54.12,67.62) | 0.07(0.07,0.08) | 32.16(29.64,34.60) | 0.26(0.23,0.30) | 107.23(94.76,119.20) |
| **Caribbean** | 3.61(2.45,5.20) | 1485.97(1008.31,2136.92) | 1.12(0.76,1。57) | 452.88(307.45,636.02) | 11.07(8.34,14.53) | 4278.48(3209.62,5612.04) | 0.37(0.31,0.43) | 141.59(118.09,165.87) | 0.07(0.06,0.09) | 26.86(22.83,32.64) | 0.31(0.27,0.36) | 110.55(95.94,127.31) |
| **Central Asia** | 2.38(1.63,3.33) | 483.56(330.71,678.72) | 2.79(1.95,3.87) | 553.49(386.57,768.61) | 25.41(19.09,32.58) | 4956.97(3739.86,6335.94) | 0.37(0.32,0.41) | 70.78(61.74,79.91) | 0.12(0.10,0.14) | 24.01(20.78,27.37) | 0.35(0.31,0.40) | 69.70(61.51,78.91) |
| **Central Europe** | 1.19(0.82,1.69) | 228.93(157.78,326.20) | 2.60(1.83,3.54) | 481.88(341.08,669.66) | 20.81(16.14,25.97) | 2874.02(2232.88,3584.04) | 0.71(0.63,0.78) | 90.48(80.59,100.41) | 0.40(0.37,0.43) | 43.47(39.98,47.08) | 1.64(1.47,1.79) | 152.72(137.41,167.81) |
| **Central Latin America** | 40.73(28.52,56.62) | 2967.64(2076.22,4124.04) | 5.76(3.98,8.04) | 413.67(285.58,577.79) | 70.43(53.56,90.04) | 4989.57(3806.85,6381.87) | 2.16(1.85,2.49) | 154.03(131.84,177.48) | 0.49(0.42,0.55) | 34.93(30.06,39.55) | 0.66(0.58,0.76) | 47.31(41.23,54.12) |
| **Central Sub-Saharan Africa** | 4.44(3.05,6.41) | 673.86(465.36,968.69) | 4.06(2.79,5.63) | 655.31(450.47,912.38) | 15.29(11.38,20.13) | 3040.03(2283.84,4001.45) | 0.63(0.41,0.86) | 133.67(90.05,182.02) | 0.06(0.04,0.09) | 14.60(8.47,20.94) | 0.09(0.06,0.13) | 24.62(15.95,35.55) |
| **East Asia** | 104.90(74.23,148.09) | 1548.43(1085.52,2170.68) | 31.16(22.68,41.21) | 409.56(292.82,543.04) | 134.37(103.27,173.01) | 1493.93(1141.77,1930.89) | 7.75(5.62,10.10) | 79.16(57.42,102.70) | 1.93(1.44,2.51) | 19.58(14.56,25.49) | 5.28(3.92,7.26) | 47.38(34.86,64.97) |
| **Eastern Europe** | 2.66(1.85,3.82) | 264.99(182.67,381.43) | 8.83(6.05,12.23) | 862.50(601.92,1189.75) | 95.92(73.28,125.03) | 6934.45(5230.48,8997.04) | 1.47(1.31,1.61) | 104.48(93.46,115.16) | 0.68(0.60,0.77) | 41.71(36.90,47.34) | 4.37(3.94,4.83) | 226.61(203.89,251.64) |
| **Eastern Sub-Saharan Africa** | 14.39(10.01,20.63) | 669.17(472.35,949.85) | 11.49(7.79,16.22) | 571.18(385.90,794.56) | 36.84(27.76,4826) | 2327.56(1749.64,3025.59) | 1.72(1.37,2.25) | 117.66(94.45,152.15) | 0.35(0.24,0.44) | 25.41(17.53,31.71) | 0.25(0.18,0.35) | 24.30(17.63,33.71) |
| **High-income Asia Pacific** | 41.05(29.23,57.75) | 5237.62(3779.21,7307.15) | 5.45(3.94,7.04) | 639.00(460.70,840.54) | 34.91(26.79,44.57) | 3502.52(2685.33,4493.03) | 0.98(0.90,1.06) | 79.24(73.58,85.98) | 0.50(0.44,0.53) | 33.22(30.01,35.16) | 1.01(0.92,1.10) | 65.55(60.19,71.27) |
| **High-income North America** | 63.62(47.43,83.24) | 3729.48(2777.89,4876.97) | 5.94(4.43,7.61) | 331.84(247.20,429.60) | 64.85(48.87,83.84) | 3033.16(2281.36,3957.99) | 1.91(1.83,1.98) | 86.28(82.71,89.75) | 1.07(1.01,1.12) | 38.38(36.76,39.93) | 8.09(7.57,8.47) | 252.73(239.06,263.63) |
| **North Africa and Middle East** | 66.73(46.72,94.35) | 2075.28(1453.41,2932.69) | 22.00(15.28,30.83) | 684.52(476.30,959.61) | 42.68(31.65,56.78) | 1368.71(1025.08,1810.34) | 0.59(0.50,0.69) | 19.29(16.43,22.81) | 0.54(0.43,0.63) | 19.50(15.67,22.61) | 1.31(0.96,1.56) | 51.70(37.60,61.50) |
| **Oceania** | 1.24(0.87,1.78) | 1772.65(1232.67,2531.47) | 0.68(0.46,0.94) | 996.78(673.44,1378.56) | 0.95(0.71,1.25) | 1550.37(1182.17,2044.04) | 0.06(0.05,0.09) | 109.68(85.14,162.49) | 0.01(0.00,0.01) | 15.47(8.78,21.50) | 0.02(0.01,0.04) | 53.87(31.01,81.42) |
| **South Asia** | 112.91(79.50,158.33) | 1135.87(799.70,1591.05) | 58.66(40.81,81.59) | 591.56(411.13,823.86) | 310.26(231.12,412.27) | 3384.79(2527.93,4468.71) | 5.72(4.94,6.58) | 63.62(54.88,73.03) | 1.93(1.61,2.31) | 22.03(18.49,26.24) | 1.56(1.31,2.08) | 19.13(16.08,25.37) |
| **Southeast Asia** | 105.20(73.79,148.10) | 2842.65(1993.15,3997.51) | 24.21(17.14,32.95) | 640.10(454.04,873.50) | 63.65(48.51,81.82) | 1641.67(1253.93,2108.75) | 2.94(2.48,3.44) | 75.11(63.34,87.74) | 1.67(1.23,2.17) | 43.52(32.08,56.58) | 1.85(1.25,2.25) | 47.19(32.15,57.57) |
| **Southern Latin America** | 6.68(4.69,9.57) | 1892.50(1326.86,2707.18) | 1.59(1.18,2.06) | 435.46(321.19,564.02) | 12.62(9.38,16.77) | 3251.72(2405.37,4329.64) | 0.51(0.47,0.56) | 132.90(121.50,145.38) | 0.13(0.12,0.14) | 31.73(29.30,33.94) | 0.23(0.21,0.26) | 51.14(46.17,56.95) |
| **Southern Sub-Saharan Africa** | 4.80(3.29,6.80) | 1094.76(749.33,1548.05) | 2.59(1.76,3.61) | 589.89(402.44,818.93) | 25.79(19.34,34.02) | 6223.91(4683.93,8138.92) | 0.70(0.60,0.81) | 172.03(147.39,197.69) | 0.10(0.08,0.12) | 26.13(20.12,30.57) | 0.14(0.11,0.17) | 39.03(29.71,46.20) |
| **Tropical Latin America** | 7.46(5.15,10.57) | 610.00(419.25,869.52) | 6.24(4.32,8.64) | 495.84(343.85,687.14) | 25.79(19.34,34.02) | 3120.36(2379.43,3983.68) | 1.48(1.41,1.56) | 109.51(104.02,115.29) | 0.36(0.34,0.37) | 26.41(24.97,27.71) | 0.69(0.64,0.73) | 48.48(45.21,51.39) |
| **Western Europe** | 74.56(52.32,104.61) | 3942.92(2761.02,5529.65) | 9.01(6.54,12.03) | 463.14(332.86,621.62) | 10.84(8.34,14.01) | 4088.41(3090.51,5352.23) | 1.65(1.56,1.73) | 59.03(56.44,61.83) | 1.46(1.36,1.53) | 40.81(38.95,42.49) | 5.94(5.43,6.28) | 143.09(133.28,150.66) |
| **Western Sub-Saharan Africa** | 17.42(12.10,24.93) | 722.68(508.32,1026.25) | 16.21(11.05,22.53) | 714.44(487.69,973.36) | 55.93(42.06,73.13) | 3099.78(2340.60,4077.08) | 1.45(1.09,1.80) | 83.68(63.82,102.81) | 0.18(0.12,0.24) | 11.78(7.92,15.22) | 0.24(0.19,0.31) | 20.39(15.67,25.96) |

**Table S5.** Regional mortality and ASMR of the six gynecological diseases in 2021.

| **Location** | **PCOS** |  | **Endometriosis** |  | **Uterine fibroids** |  | **Cervical cancer** |  | **Ovarian cancer** |  | **Uterine cancer** |  |
| --- | --- | --- | --- | --- | --- | --- | --- | --- | --- | --- | --- | --- |
|  | **Deaths (, 95% UI)** | **ASMR (1/100,000, 95% UI)** | **Deaths (95% UI)** | **ASMR (1/100,000, 95% UI)ASMR (95% UI)** | **Deaths (, 95% UI)** | **ASMR (1/100,000, 95% UI)** | **Deaths (× 105, 95% UI)** | **ASMR (1/100,000, 95% UI)** | **Deaths (× 105, 95% UI)** | **ASMR (1/100,000, 95% UI)** | **Deaths (× 105, 95% UI)** | **ASMR (1/100,000, 95% UI)** |
| **Global** | 0 | 0 | 54.17(21.51,121.92) | 0.00(0.00,0.00) | 2077.75(1225.41,2574.28) | 0.05(0.03,0.06) | 2.97(2.72,3.22) | 6.62(6.07,7.18) | 1.86(1.68,2.01) | 4.06(3.67,4.40) | 0.98(0.87,1.08) | 2.11(1.87,2.34) |
| **High SDI** | 0 | 0 | 5.90(2.91,8.61) | 0.00(0.00,0.00) | 119.49(102.66,137.46) | 0.01(0.01,0.02) | 0.26(0.23,0.27) | 2.62(2.44,2.74) | 0.56(0.50,0.60) | 5.08(4.62,5.37) | 0.32(0.28,0.34) | 2.69(2.41,2.84) |
| **High-middle SDI** | 0 | 0 | 8.60(3.45,15.30) | 0.00(0.00,0.00) | 290.00(196.46,372.42) | 0.03(0.02,0.04) | 0.46(0.41,0.53) | 4.59(4.02,5.20) | 0.44(0.39,0.49) | 4.16(3.68,4.63) | 0.26(0.23,0.29) | 2.39(2.13,2.66) |
| **Middle SDI** | 0 | 0 | 24.12(8.94,49.48) | 0.00(0.00,0.00) | 711.47(376.14,951.24) | 0.05(0.03,0.07) | 0.96(0.87,1.064) | 6.72(6.05,7.43) | 0.46(0.40,0.51) | 3.18(2.78,3.57) | 0.23(0.19,0.27) | 1.61(1.35,1.87) |
| **Low-middle SDI** | 0 | 0 | 11.25(4.31,33.61) | 0.00(0.00,0.00) | 648.36(391.90,871.36) | 0.09(0.05,0.11) | 0.78(0.70,0.86) | 9.71(8.67,10.70) | 0.29(0.25,0.34) | 3.72(3.24,4.40) | 0.12(0.10,0.15) | 1.64(1.42,2.07) |
| **Low SDI** | 0 | 0 | 4.26(0.62,27.62) | 0.00(0.00,0.01) | 306.86(121.08,480.29) | 0.09(0.04,0.14) | 0.50(0.42,0.51) | 16.36(13.94,19.38) | 0.10(0.08,0.12) | 3.74(2.87,4.43) | 0.05(0.04,0.06) | 1.83(1.46,2.36) |
| **Andean Latin America** | 0 | 0 | 0.18(0.05,0.72) | 0.00(0.00,0.00) | 17.25(12.77,26.03) | 0.05(0.04,0.08) | 0.04(0.03,0.06) | 14.02(10.85,17.51) | 0.01(0.01,0.02) | 4.22(3.27,5.26) | 0.01(0.01,0.01) | 3.17(2.37,4.10) |
| **Australasia** | 0 | 0 | 0.10(0.01,0.25) | 0.00(0.00,0.00) | 0.48(0.38,0.61) | 0.00(0.00,0.00) | 0.00(0.00,0.01) | 1.85(1.66,2.02) | 0.01(0.01,0.01) | 4.68(4.12,5.08) | 0.01(0.01,0.01) | 2.12(1.78,2.39) |
| **Caribbean** | 0 | 0 | 0.31(0.12,0.89) | 0.00(0.00,0.00) | 34.90(25.32,49.56) | 0.13(0.10,0.19) | 0.03(0.03,0.04) | 12.31(10.33,14.68) | 0.01(0.01,0.01) | 4.17(3.59,4.95) | 0.02(0.01,0.02) | 5.38(4.63,6.24) |
| **Central Asia** | 0 | 0 | 0.23(0.07,0.67) | 0.00(0.00,0.00) | 33.28(27.26,40.71) | 0.07(0.05,0.08) | 0.03(0.003,0.03) | 6.33(5.62,7.13) | 0.02(0.02,0.02) | 4.08(3.57,4.63) | 0.01(0.01,0.01) | 2.68(2.40,3.01) |
| **Central Europe** | 0 | 0 | 0.39(0.12,0.72) | 0.00(0.00,0.00) | 7.17(5.85,10.00) | 0.01(0.01,0.01) | 0.07(0.06,0.07) | 6.02(5.53,6.53) | 0.09(0.08,0.10) | 7.40(6.79,7.96) | 0.06(0.05,0.06) | 4.17(3.77,4.57) |
| **Central Latin America** | 0 | 0 | 2.44(1.22,5.88) | 0.00(0.00,0.00) | 96.26(83.27,110.25) | 0.07(0.06,0.08) | 0.13(0.11,0.15) | 9.52(8.21,10.86) | 0.07(0.06,0.08) | 4.90(4.32,5.50) | 0.03(0.03,0.03) | 2.15(1.88,2.42) |
| **Central Sub-Saharan Africa** | 0 | 0 | 0.53(0.08,3.60) | 0.00(0.00,0.01) | 49.55(5.61,99.60) | 0.12(0.01,0.26) | 0.09(0.06,0.12) | 25.10(17.45,33.97) | 0.01(0.001,0.01) | 3.10(1.69,4.42) | 0.01(0.00,0.01) | 2.14(1.41,3.16) |
| **East Asia** | 0 | 0 | 4.63(0.58,11.01) | 0.00(0.00,0.00) | 485.20(179.71,679.89) | 0.04(0.02,0.06) | 0.52(0.39,0.67) | 4.68(3.55,5.98) | 0.26(0.20,0.34) | 2.33(1.72,3.01) | 0.14(0.11,0.19) | 1.25(0.93,1.69) |
| **Eastern Europe** | 0 | 0 | 4.35(2.01,7.22) | 0.00(0.00,0.01) | 78.41(67.47,93.01) | 0.05(0.04,0.06) | 0.10(0.09,0.11) | 5.50(4.96,6.07) | 0.12(0.11,0.14) | 6.00(5.37,6.73) | 0.10(0.09,0.11) | 4.65(4.18,5.20) |
| **Eastern Sub-Saharan Africa** | 0 | 0 | 3.24(0.54,17.71) | 0.00(0.00,0.01) | 36.03(5.47,100.59) | 0.03(0.00,0.07) | 0.23(0.19,0.29) | 21.68(17.79,26.85) | 0.05(0.04,0.06) | 5.21(3.74,6.35) | 0.02(0.01,0.03) | 2.17(1.59,2.95) |
| **High-income Asia Pacific** | 0 | 0 | 0.50(0.15,1.08) | 0.00(0.00,0.00) | 34.28(27.57,40.13) | 0.02(0.01,0.02) | 0.05(0.04,0.06) | 2.53(2.28,2.73) | 0.07(0.06,0.08) | 3.26(2.87,3.50) | 0.04(0.03,0.04) | 1.65(1.44,1.80) |
| **High-income North America** | 0 | 0 | 4.13(2.10,5.94) | 0.00(0.00,0.00) | 37.81(34.31,41.99) | 0.02(0.01,0.02) | 0.08(0.07,0.08) | 2.64(2.50,2.75) | 0.19(0.17,0.20) | 5.44(4.96,5.71) | 0.13(0.12,0.14) | 3.57(3.23,3.77) |
| **North Africa and Middle East** | 0 | 0 | 0.54(0.10,3.58) | 0.00(0.00,0.00) | 56.59(36.49,98.22) | 0.02(0.01,0.04) | 0.06(0.05,0.07) | 2.55(2.17,2.93) | 0.07(0.06,0.08) | 3.14(2.54,3.65) | 0.03(0.02,0.03) | 1.34(1.01,1.60) |
| **Oceania** | 0 | 0 | 0.05(0.01,0.41) | 0.00(0.00,0.01) | 1.96(0.69,4.05) | 0.04(0.01,0.07) | 0.01(0.01,0.01) | 15.27(12.10,22.32) | 0.00(0.00,0.00) | 1.97(1.23,2.64) | 0.00(0.00,0.00) | 3.55(2.06,5.32) |
| **South Asia** | 0 | 0 | 5.38(0.51,31.30) | 0.00(0.00,0.00) | 632.86(382.44,987.05) | 0.09(0.05,0.13) | 0.70(0.61,0.80) | 8.72(7.57,9.88) | 0.31(0.26,0.36) | 3.92(3.39,4.67) | 0.09(0.08,0.12) | 1.24(1.03,1.66) |
| **Southeast Asia** | 0 | 0 | 14.45(4.92,34.37) | 0.00(0.00,0.01) | 60.75(10.64,111.58) | 0.02(0.00,0.03) | 0.28(0.24,0.32) | 7.45(6.43,8.59) | 0.15(0.11,0.19) | 3.96(3.07,5.12) | 0.07(0.05,0.09) | 2.03(1.47,2.47) |
| **Southern Latin America** | 0 | 0 | 0.39(0.05,0.79) | 0.00(0.00,0.00) | 5.21(4.44,6.21) | 0.01(0.01,0.01) | 0.04(0.04,0.04) | 8.56(7.89,9.28) | 0.02(0.02,0.02) | 4.82(4.44,5.17) | 0.01(0.01,0.01) | 2.00(1.79,2.18) |
| **Southern Sub-Saharan Africa** | 0 | 0 | 0.75(0.31,1.96) | 0.00(0.00,0.00) | 71.37(43.40,91.77) | 0.19(0.11,0.25) | 0.09(0.07,0.10) | 23.90(21.02,26.67) | 0.02(0.01,0.02) | 5.38(4.05,6.16) | 0.01(0.01,0.01) | 2.94(2.15,3.41) |
| **Tropical Latin America** | 0 | 0 | 10.01(3.38,15.25) | 0.01(0.00,0.01) | 85.56(78.31,93.02) | 0.06(0.06,0.07) | 0.12(0.11,0.12) | 8.38(7.82,8.86) | 0.06(0.05,0.06) | 3.97(3.67,4.20) | 0.03(0.03,0.04) | 2.40(2.19,2.57) |
| **Western Europe** | 0 | 0 | 0.83(0.45,3.07) | 0.00(0.00,0.00) | 34.18(29.38,38.82) | 0.01(0.01,0.01) | 0.10(0.09,0.11) | 2.29(2.11,2.43) | 0.28(0.25,0.30) | 5.55(5.03,5.90) | 0.14(0.12,0.15) | 2.53(2.23,2.71) |
| **Western Sub-Saharan Africa** | 0 | 0 | 0.76(0.03,10.27) | 0.00(0.00,0.01) | 218.66(52.17,352.89) | 0.14(0.03,0.22) | 0.19(0.15,0.23) | 15.57(12.55,18.53) | 0.03(0.02,0.04) | 2.58(1.69,3.28) | 0.02(0.01,0.02) | 1.90(1.48,2.37) |

**Table S6.** Regional DALYs and ASDR for six gynecological diseases in 2021.

| **Location** | **PCOS** |  | **Endometriosis** |  | **Uterine fibroids** |  | **Cervical cancer** |  | **Ovarian cancer** |  | **Uterine cancer** |  |
| --- | --- | --- | --- | --- | --- | --- | --- | --- | --- | --- | --- | --- |
|  | **DALYs (× 105, 95% UI)** | **ASDR**  **(1/100,000, 95% UI)** | **DALYs (× 105, 95% UI)** | **ASDR**  **(1/100,000, 95% UI)** | **DALYs (× 105, 95% UI)** | **ASDR**  **(1/100,000, 95% UI)** | **DALYs (× 105, 95% UI)** | **ASDR**  **(1/100,000, 95% UI)** | **DALYs (× 105, 95% UI)** | **ASDR**  **(1/100,000, 95% UI)** | **DALYs (× 105, 95% UI)** | **ASDR**  **(1/100,000, 95% UI)** |
| **Global** | 6.08(2.73,12.69) | 15.40(6.91,32.13) | 20.49(11.95,31.34) | 51.27(29.87,78.43) | 1.43(1.02,1.93) | 3.39(2.43,4.59) | 99.12(90.53,107.98) | 226.28(206.51,246.86) | 51.63(46.92,56.08) | 115.15(104.58,125.21) | 25.63(22.91,28.46) | 56.15(50.07,62.37) |
| **High SDI** | 1.54(0.71,3.15) | 31.37(14.37,64.00) | 2.30(1.37,3.54) | 43.84(26.40,66.59) | 0.11(0.08,0.18) | 1.69(1.11,2.69) | 7.08(6.65,7.42) | 86.41(82.45,90.30) | 12.76(11.73,13.43) | 133.80(125.32,140.06) | 7.35(6.68,7.83) | 71.79(66.24,76.26) |
| **High-middle SDI** | 0.97(0.43,2.05) | 15.91(7.10,33.31) | 3.15(1.87,4.94) | 47.57(28.31,72.59) | 0.20(0.14,0.28) | 2.36(1.66,3.37) | 14.32(12.53,16.35) | 152.90(133.88,174.32) | 11.94(10.55,13.36) | 119.79(105.13,134.14) | 6.88(6.19,7.67) | 65.74(58.93,73.57) |
| **Middle SDI** | 2.15(0.96,4.51) | 17.26(7.68,36.24) | 6.04(3.56,9.31) | 46.77(27.46,71.92) | 0.44(0.30,0.60) | 3.18(2.15,4.32) | 31.67(28.57,35.10) | 218.95(197.60,242.31) | 14.16(12.44,15.87) | 97.76(85.77,109.48) | 6.57(5.37,7.70) | 45.05(36.88,52.70) |
| **Low-middle SDI** | 1.07(0.47,2.24) | 10.43(4.56,21.89) | 5.59(3.23,8.69) | 55.32(32.22,85.34) | 0.45(0.32,0.60) | 4.92(3.55,6.51) | 27.51(24.55,30.57) | 321.36(287.11,356.77) | 9.26(8.02,10.91) | 111.40(96.54,131.20) | 3.47(2.95,4.30) | 43.77(37.41,54.37) |
| **Low SDI** | 0.34(0.15,0.73) | 6.20(2.67,13.05) | 3.40(1.94,5.29) | 65.33(37.51,100.53) | 0.22(0.13,0.31) | 5.36(3.09,7.56) | 18.43(15.52,22.00) | 535.11(454.02,638.34) | 3.45(2.60,4.13) | 108.72(82.67,129.40) | 1.32(1.05,1.70) | 46.80(37.40,60.25) |
| **Andean Latin America** | 0.10(0.044,0.21) | 28.78(12.57,60.15) | 0.14(0.08,0.22) | 40.13(23.00,62.35) | 0.02(0.01,0.02) | 4.73(3.33,6.85) | 1.40(1.08,1.77) | 431.74(331.78,543.15) | 0.40(0.31,0.51) | 125.60(95.71,158.01) | 0.26(0.19,0.34) | 82.94(61.69,107.41) |
| **Australasia** | 0.06(0.03,0.13) | 41.77(18.75,86.35) | 0.07(0.04,0.12) | 49.52(29.15,77.73) | 0.00(0.00,0.00) | 0.42(0.24,0.76) | 0.13(0.12,0.15) | 61.68(56.34,67.50) | 0.29(0.26,0.32) | 116.72(106.15,125.99) | 0.14(0.12,0.16) | 53.92(47.26,60.21) |
| **Caribbean** | 0.03(0.01,0.07) | 13.03(5.70,27.37) | 0.10(0.06,0.16) | 41.52(23.82,63.65) | 0.02(0.02,0.03) | 8.05(5.91,11.10) | 1.16(0.96,1.41) | 433.17(356.80,526.53) | 0.34(0.29,0.42) | 124.35(106.00,151.66) | 0.40(0.35,0.47) | 144.02(122.94,168.01) |
| **Central Asia** | 0.02(0.01,0.04) | 4.24(1.81,9.10) | 0.26(0.15,0.40) | 51.22(30.55,79.31) | 0.03(0.02,0.04) | 5.71(4.28,7.79) | 1.08(0.95,1.23) | 213.84(187.54,241.95) | 0.62(0.54,0.71) | 124.11(108.08,141.31) | 0.37(0.32,0.42) | 75.47(66.42,85.96) |
| **Central Europe** | 0.01(0.00,0.02) | 2.00(0.86,4.17) | 0.24(0.14,0.38) | 44.65(26.13,69.21) | 0.01(0.01,0.02) | 1.52(0.92,2.52) | 1.82(1.66,1.97) | 191.83(175.35,209.14) | 2.16(1.98,2.32) | 205.22(189.37,221.33) | 1.25(1.13,1.37) | 105.93(95.65,116.22) |
| **Central Latin America** | 0.35(0.16,0.74) | 25.74(11.36,53.72) | 0.53(0.31,0.82) | 38.12(21.92,58.85) | 0.06(0.05,0.08) | 4.50(3.51,6.00) | 4.42(3.81,5.09) | 315.97(272.32,363.77) | 2.10(1.83,2.38) | 150.76(131.25,170.19) | 0.81(0.70,0.93) | 58.74(50.83,66.90) |
| **Central Sub-Saharan Africa** | 0.04(0.02,0.08) | 5.82(2.51,12.02) | 0.37(0.21,0.58) | 59.69(34.10,91.76) | 0.03(0.01,0.05) | 6.62(2.41,11.34) | 3.31(2.28,4.48) | 813.59(562.84,1104.29) | 0.34(0.19,0.49) | 90.01(50.59,129.58) | 0.18(0.12,0.27) | 55.82(36.31,80.74) |
| **East Asia** | 0.90(0.39,1.86) | 13.38(5.91,27.61) | 2.90(1.70,4.60) | 38.24(22.80,60.19) | 0.19(0.10,0.26) | 1.94(1.04,2.67) | 16.16(11.95,20.80) | 151.15(111.80,195.41) | 7.87(5.81,10.25) | 72.07(53.13,94.15) | 4.25(3.20,5.71) | 38.29(28.87,51.57) |
| **Eastern Europe** | 0.02(0.01,0.05) | 2.35(0.97,4.92) | 0.81(0.47,1.27) | 79.58(46.32,123.71) | 0.08(0.05,0.11) | 5.41(3.88,7.90) | 3.20(2.89,3.54) | 200.77(180.38,221.47) | 3.32(2.964,3.72) | 180.89(160.79,203.55) | 2.70(2.43,3.03) | 133.05(118.82,149.61) |
| **Eastern Sub-Saharan Africa** | 0.12(0.05,0.26) | 5.77(2.49,12.17) | 1.06(0.60,1.63) | 52.32(29.68,80.83) | 0.04(0.02,0.07) | 2.49(1.24,4.64) | 8.77(7.02,11.06) | 709.49(572.26,890.60) | 1.70(1.18,2.12) | 150.96(106.18,185.66) | 0.53(0.38,0.75) | 54.81(39.90,75.48) |
| **High-income Asia Pacific** | 0.36(0.16,0.72) | 45.62(20.81,92.93) | 0.50(0.31,0.77) | 59.29(36.70,88.00) | 0.02(0.01,0.03) | 1.89(1.24,3.07) | 1.33(1.20,1.45) | 87.68(81.63,94.36) | 1.68(1.49,1.80) | 98.97(89.26,104.57) | 0.88(0.78,0.97) | 47.34(42.79,51.08) |
| **High-income North America** | 0.56(0.26,1.14) | 33.02(15.20,66.90) | 0.54(0.33,0.83) | 30.28(18.79,46.49) | 0.04(0.03,0.06) | 1.78(1.21,2.73) | 2.40(2.29,2.51) | 92.68(88.88,96.91) | 4.39(4.09,4.60) | 139.13(131.42,145.12) | 3.30(3.04,3.53) | 98.72(91.50,105.63) |
| **North Africa and Middle East** | 0.59(0.26,1.26) | 18.40(8.24,39.14) | 2.01(1.16,3.07) | 62.45(36.26,95.28) | 0.04(0.03,0.06) | 1.47(1.01,2.12) | 2.18(1.83,2.59) | 80.04(67.65,93.68) | 2.26(1.84,2.62) | 88.12(71.92,102.25) | 0.86(0.66,1.03) | 35.71(27.12,42.52) |
| **Oceania** | 0.01(0.00,0.02) | 15.41(6.71,32.47) | 0.06(0.04,0,10) | 91.69(52.17,145.64) | 0.00(0.00,0.00) | 2.62(1.43,4.20) | 0.25(0.19,0.38) | 499.47(390.49,739.25) | 0.03(0.02,0.04) | 59.04(35.09,80.86) | 0.04(0.02,0.07) | 100.13(56.81,152.20) |
| **South Asia** | 0.99(0.43,2.07) | 9.94(4.34,20.79) | 5.37(3.13,8.30) | 54.12(31.66,83.14) | 0.49(0.35,0.68) | 5.46(3.88,7.48) | 24.32(20.98,27.73) | 285.98(247.23,325.23) | 9.60(8.23,11.42) | 116.00(99.82,138.16) | 2.57(2.14,3.44) | 32.35(27.04,43.28) |
| **Southeast Asia** | 0.93(0.41,1.91) | 25.06(11.10,51.68) | 2.25(1.33,3.52) | 59.57(34.99,92.69) | 0.05(0.03,0.09) | 1.42(0.76,2.23) | 9.32(8.00,10.86) | 241.92(207.75,281.44) | 4.85(3.69,6.34) | 126.63(96.38,165.18) | 2.27(1.57,2.79) | 59.51(41.41,72.97) |
| **Southern Latin America** | 0.06(0.03,0.12) | 16.74(7.33,34.66) | 0.15(0.09,0.22) | 39.91(24.14,61.10) | 0.01(0.00,0.01) | 1.75(1.07,2.85) | 1.23(1.145,1.33) | 296.76(276.24,321.05) | 0.61(0.56,0.65) | 137.30(127.41,146.65) | 0.23(0.21,0.26) | 49.91(45.26,55.08) |
| **Southern Sub-Saharan Africa** | 0.04(0.02,0.09) | 9.47(4.10,19.92) | 0.23(0.13,0.37) | 53.45(30.70,82.78) | 0.04(0.03,0.06) | 10.58(7.30,13.62) | 3.01(2.61,3.39) | 788.82(685.47,885.31) | 0.54(0.42,0.63) | 150.23(114.98,174.11) | 0.25(0.190,0.29) | 72.06(54.21,84.72) |
| **Tropical Latin America** | 0.07(0.03,0.14) | 5.38(2.33,11.29) | 0.57(0.33,0.87) | 45.60(26.35,69.31) | 0.06(0.04,0.07) | 4.06(3.27,5.27) | 3.95(3.73,4.15) | 285.57(269.19,299.68) | 1.64(1.54,1.72) | 117.39(110.28,123.42) | 0.87(0.81,0.923) | 61.58(56.94,65.20) |
| **Western Europe** | 0.66(0.30,1.37) | 35.16(15.84,72.77) | 0.83(0.49,1.28) | 42.61(25.08,65.09) | 0.05(0.03,0.08) | 1.70(0.99,2.91) | 2.57(2.40,2.72) | 72.71(68.94,76.61) | 5.94(5.42,6.30) | 140.16(131.08,146.93) | 2.95(2.61,3.18) | 63.54(57.66,68.10) |
| **Western Sub-Saharan Africa** | 0.15(0.07,0.32) | 6.24(2.69,13.23) | 1.49(0.85,2.29) | 65.38(37.53,100.96) | 0.15(0.06,0.21) | 8.02(3.47,11.67) | 7.10(5.49,8.65) | 490.75(383.61,591.44) | 0.94(0.63,1.21) | 72.64(48.64,93.39) | 0.49(0.38,0.63) | 45.23(34.73,57.30) |


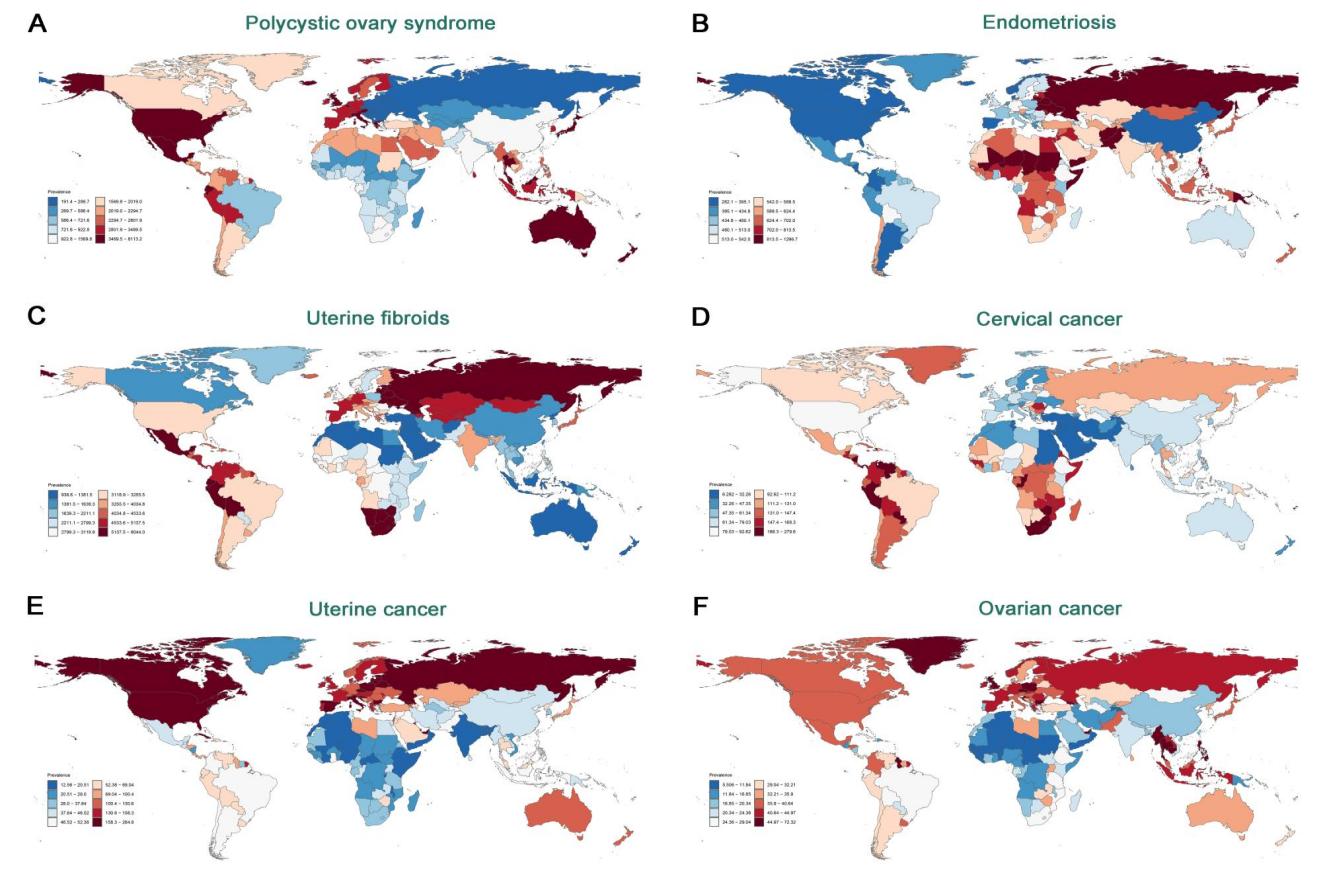


**Figure S1.** ASPR for six gynecological diseases in 2021: (A) PCOS, (B) Endometriosis, (C) Uterine Fibroids, (D) Cervical Cancer, (E) Ovarian Cancer, (F) Uterine Cancer.


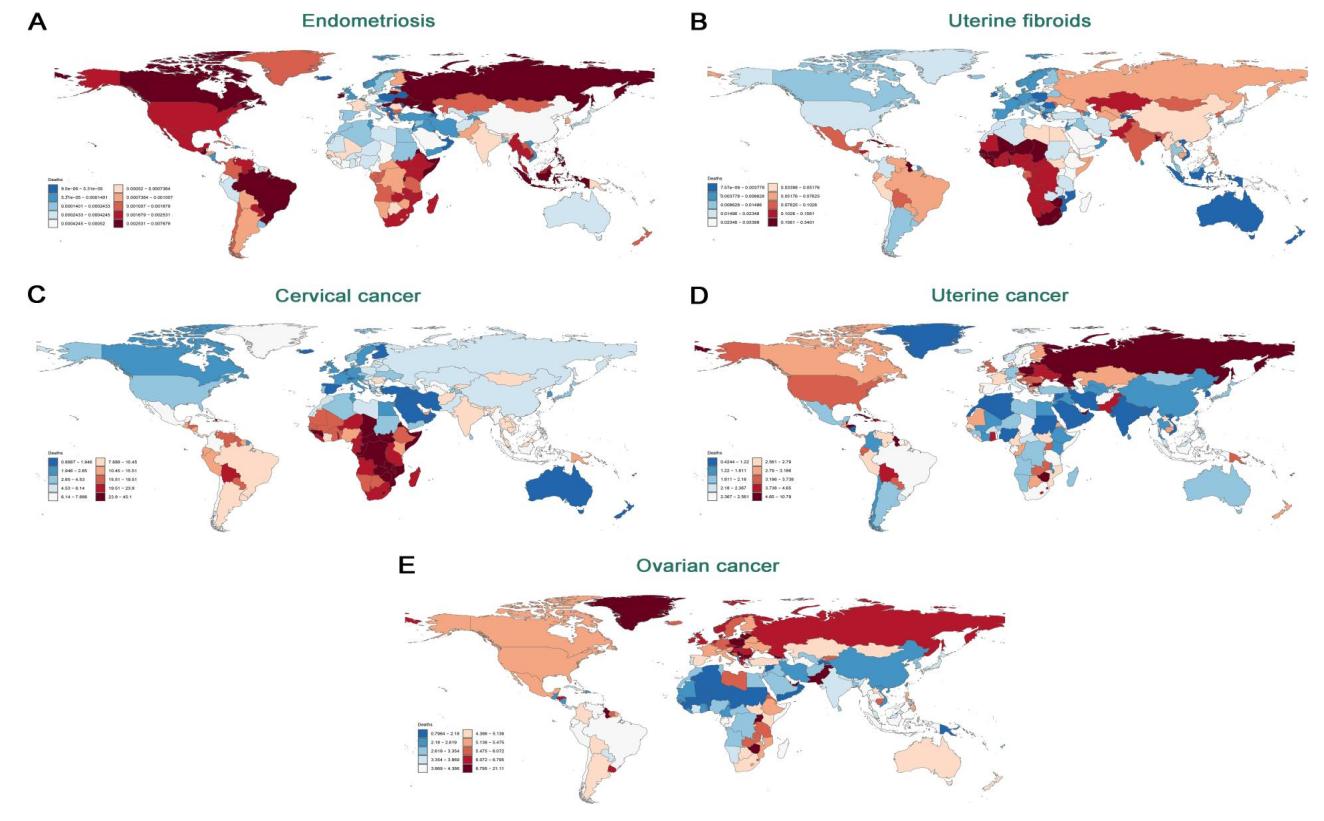


**Figure S2.** ASMR for six gynecological diseases in 2021: (A) PCOS, (B) Endometriosis, (C) Uterine Fibroids, (D) Cervical Cancer, (E) Ovarian Cancer, (F) Uterine Cancer.


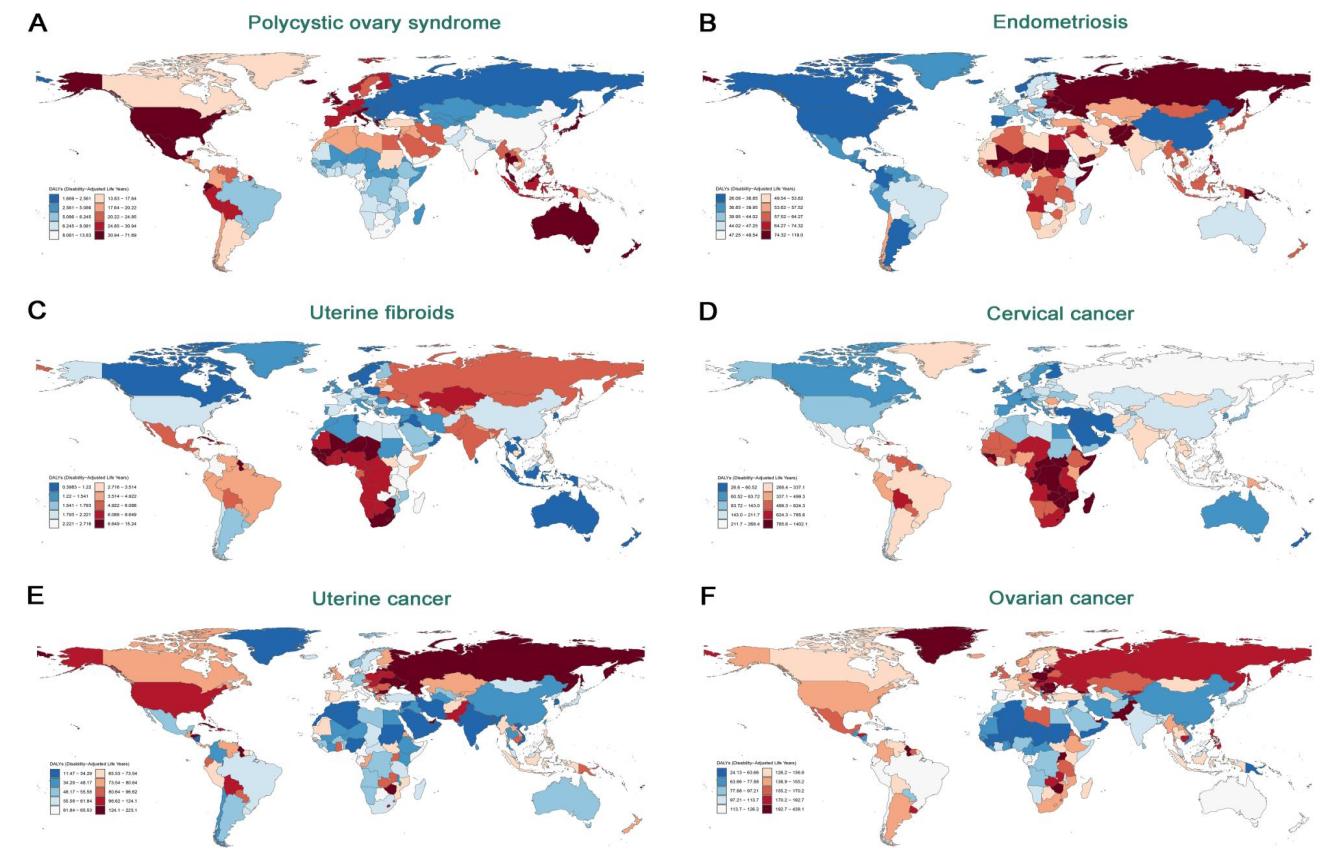


**Figure S3.** ASDR for six gynecological diseases in 2021: (A) PCOS, (B) Endometriosis, (C) Uterine Fibroids, (D) Cervical Cancer, (E) Ovarian Cancer, (F) Uterine Cancer.


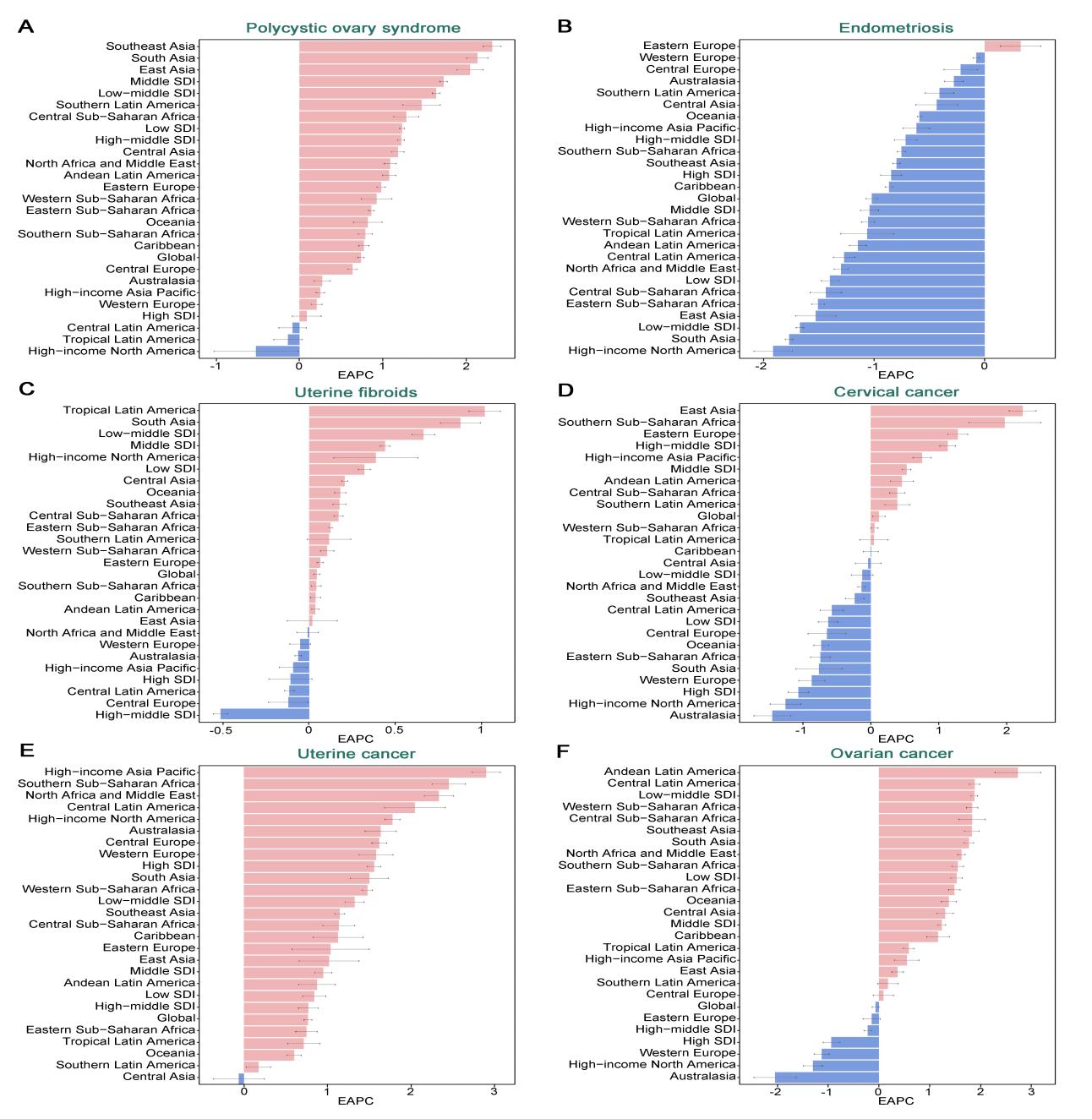


**Figure S4.** EAPC of ASPR for six gynecological diseases globally and across 21 regions.


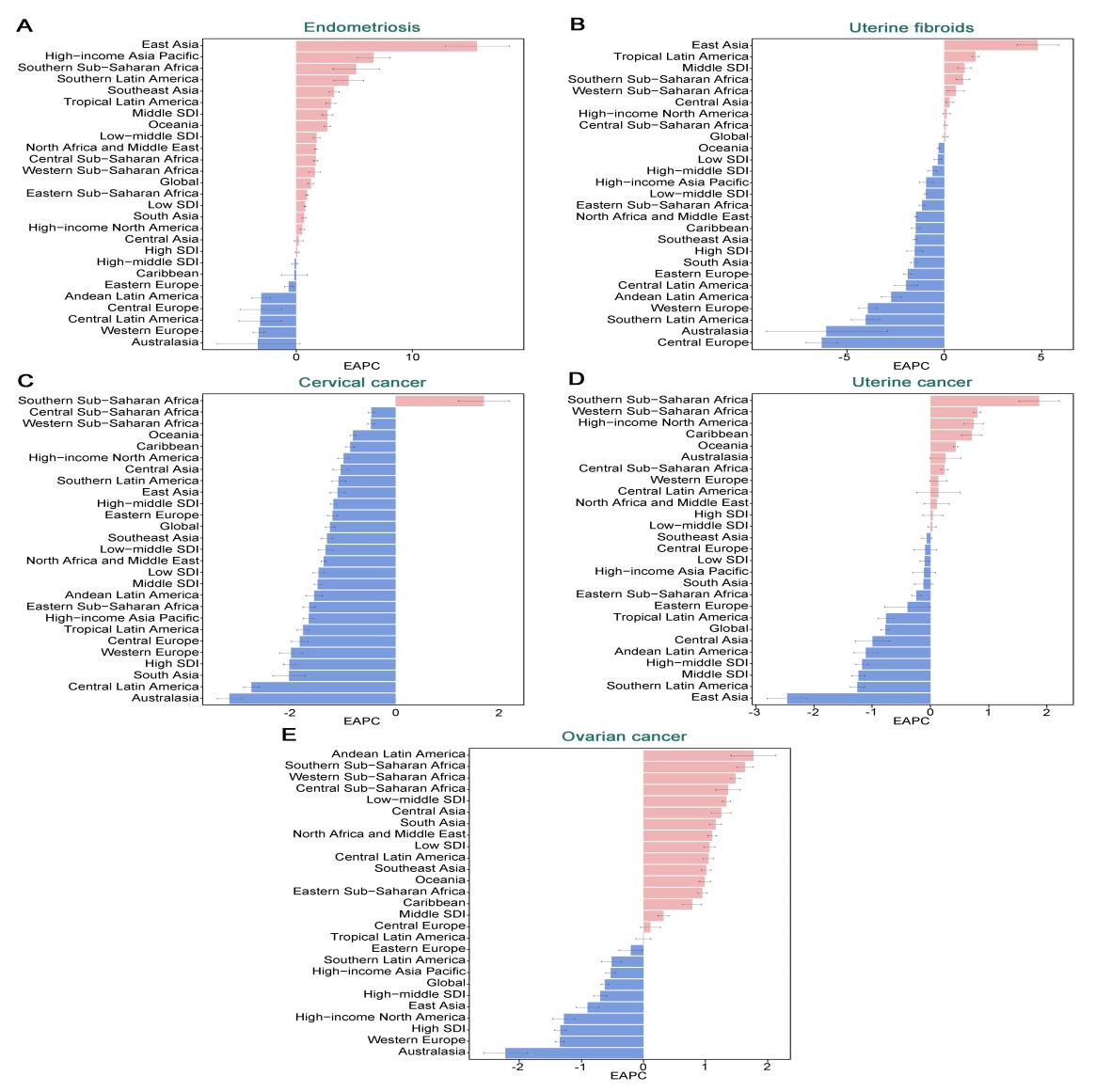


**Figure S5.** EAPC of ASMR for five gynecological diseases globally and across 21 regions.


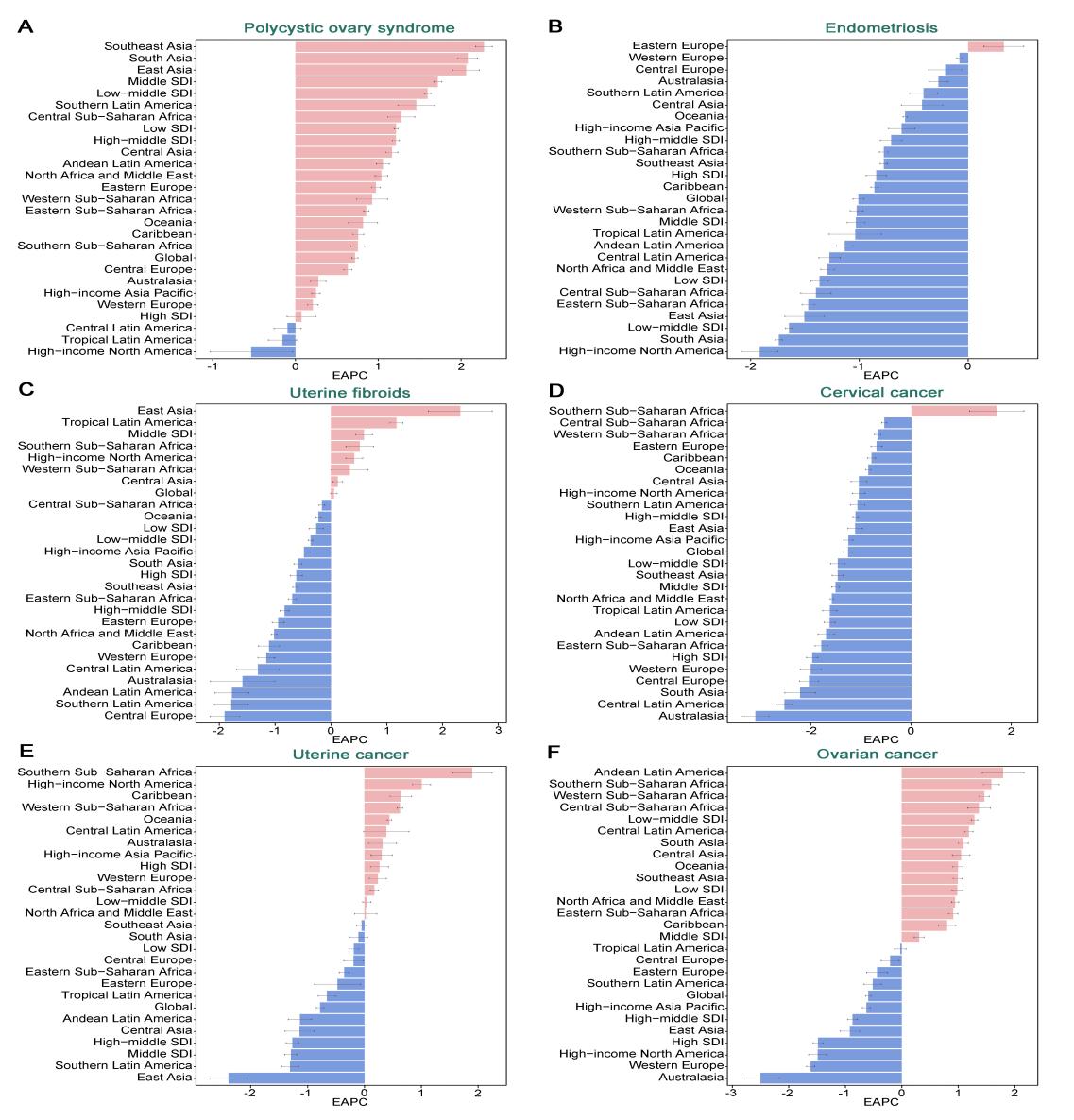


**Figure S6.** EAPC of ASDR for six gynecological diseases globally and across 21 regions.

**
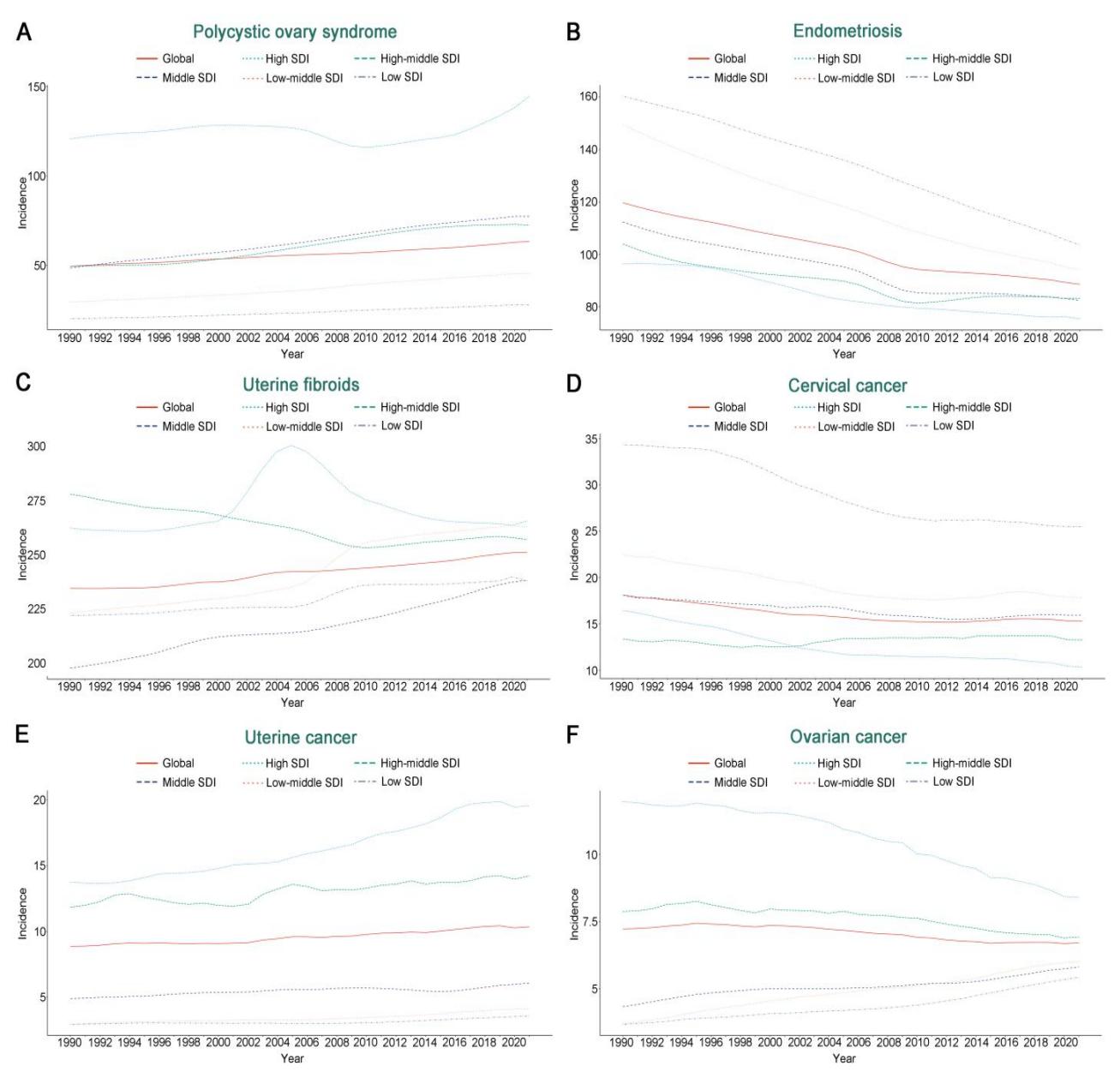
**

**Figure S7.** ASIR for six gynecological diseases in different SDI regions from 1990 to 2021.


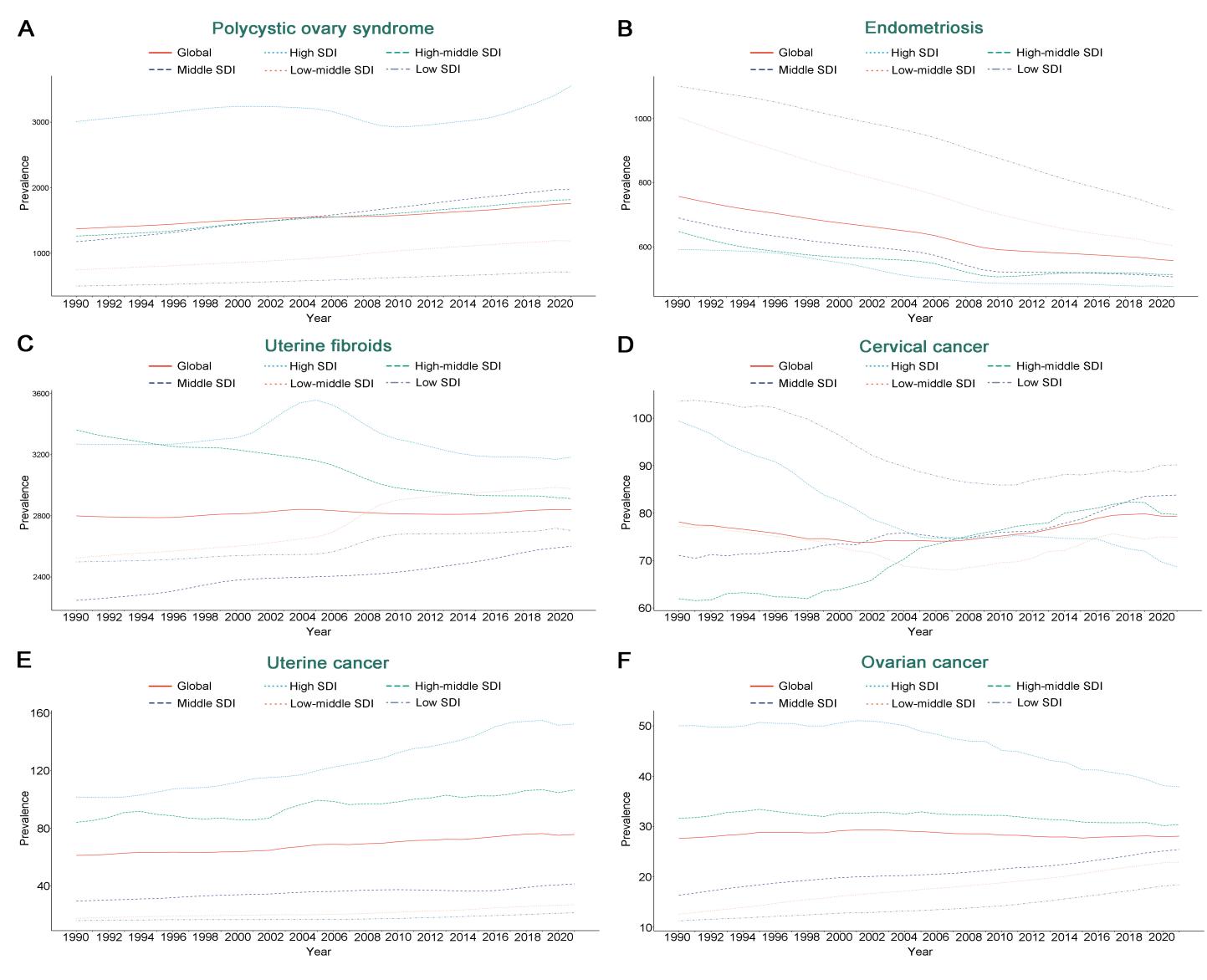


**Figure S8.** ASPR for six gynecological diseases in different SDI regions from 1990 to 2021.


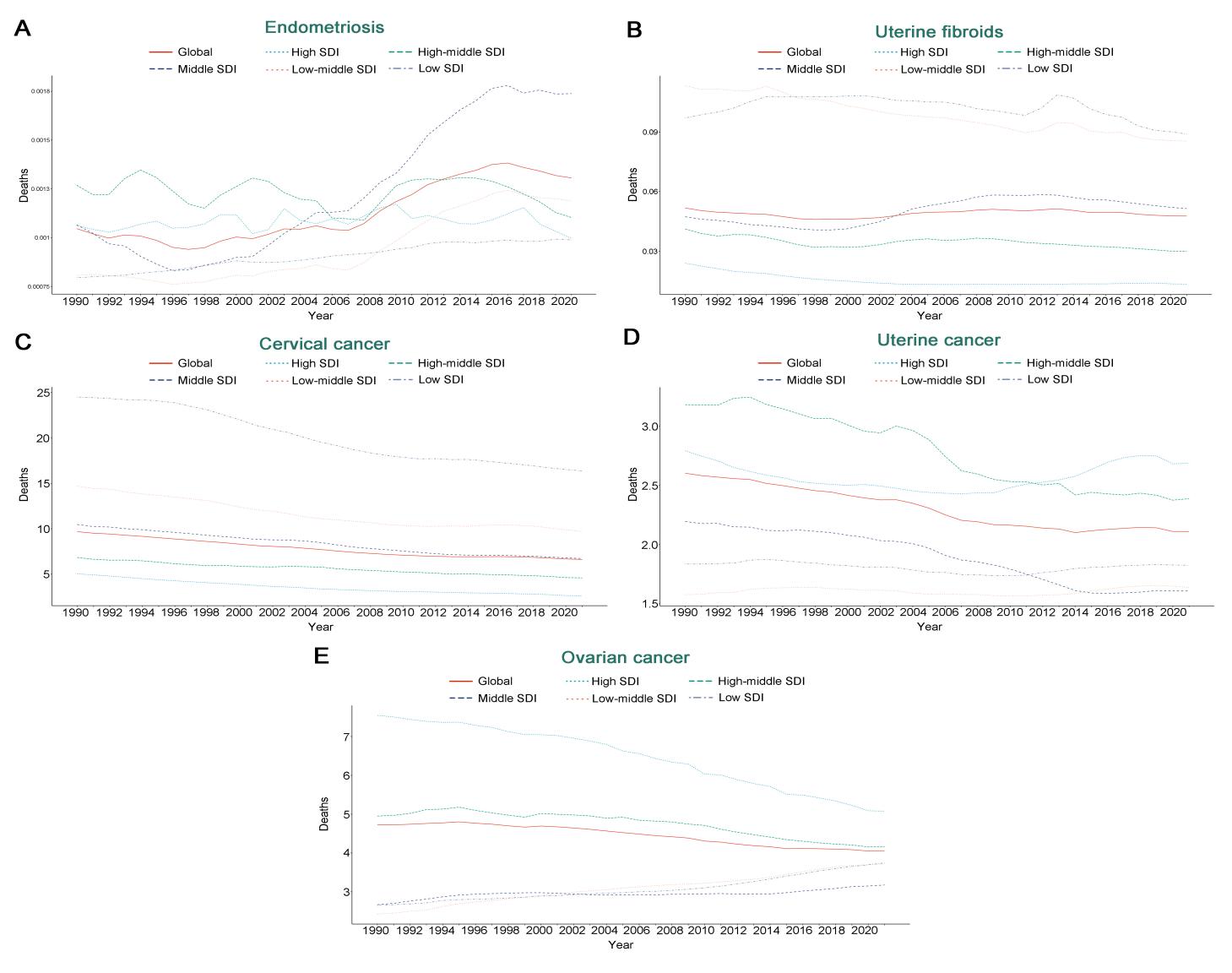


**Figure S9.** ASMR for five gynecological diseases in different SDI regions from 1990 to 2021.


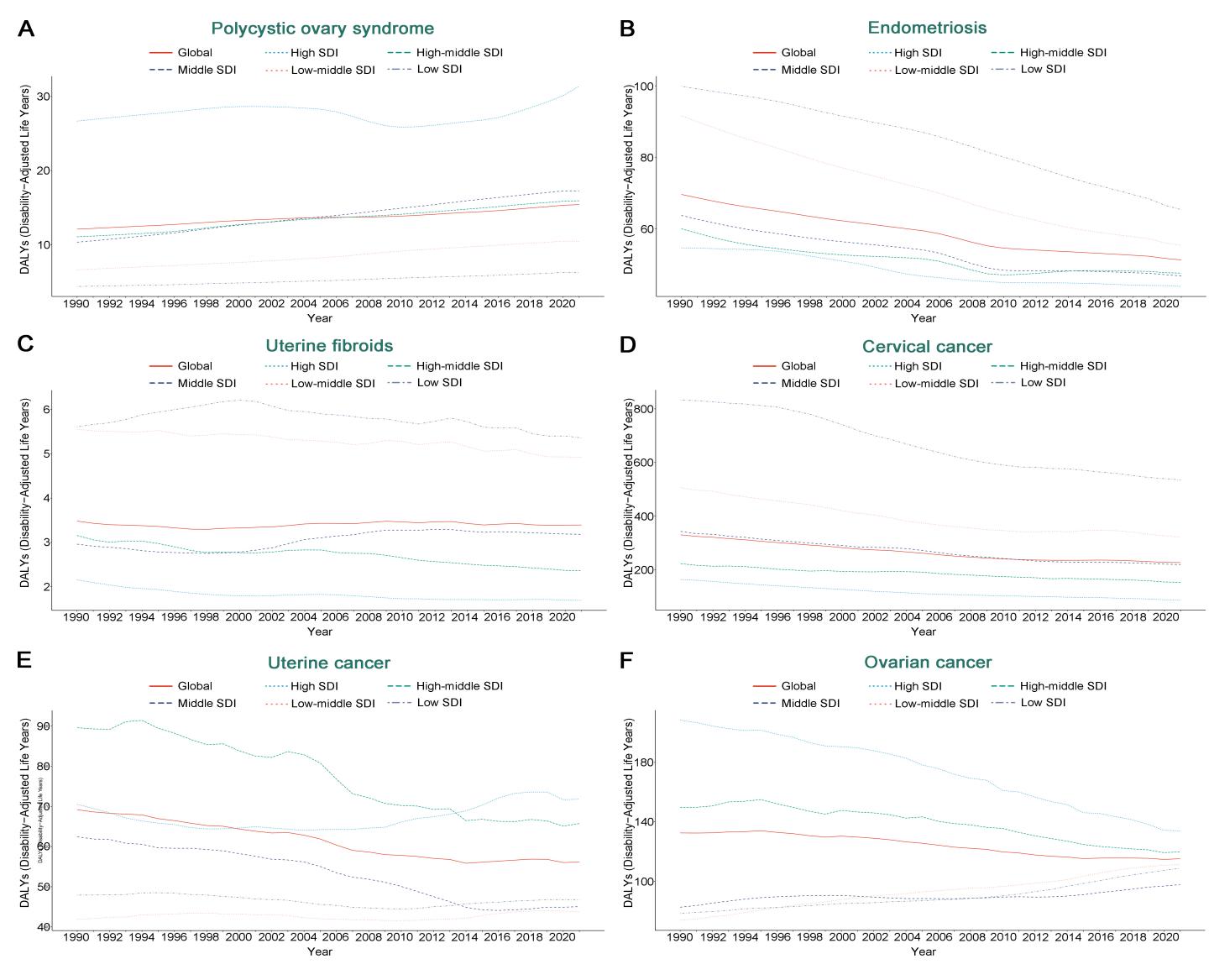


**Figure S10.** ASDR for six gynecological diseases in different SDI regions from 1990 to 2021.


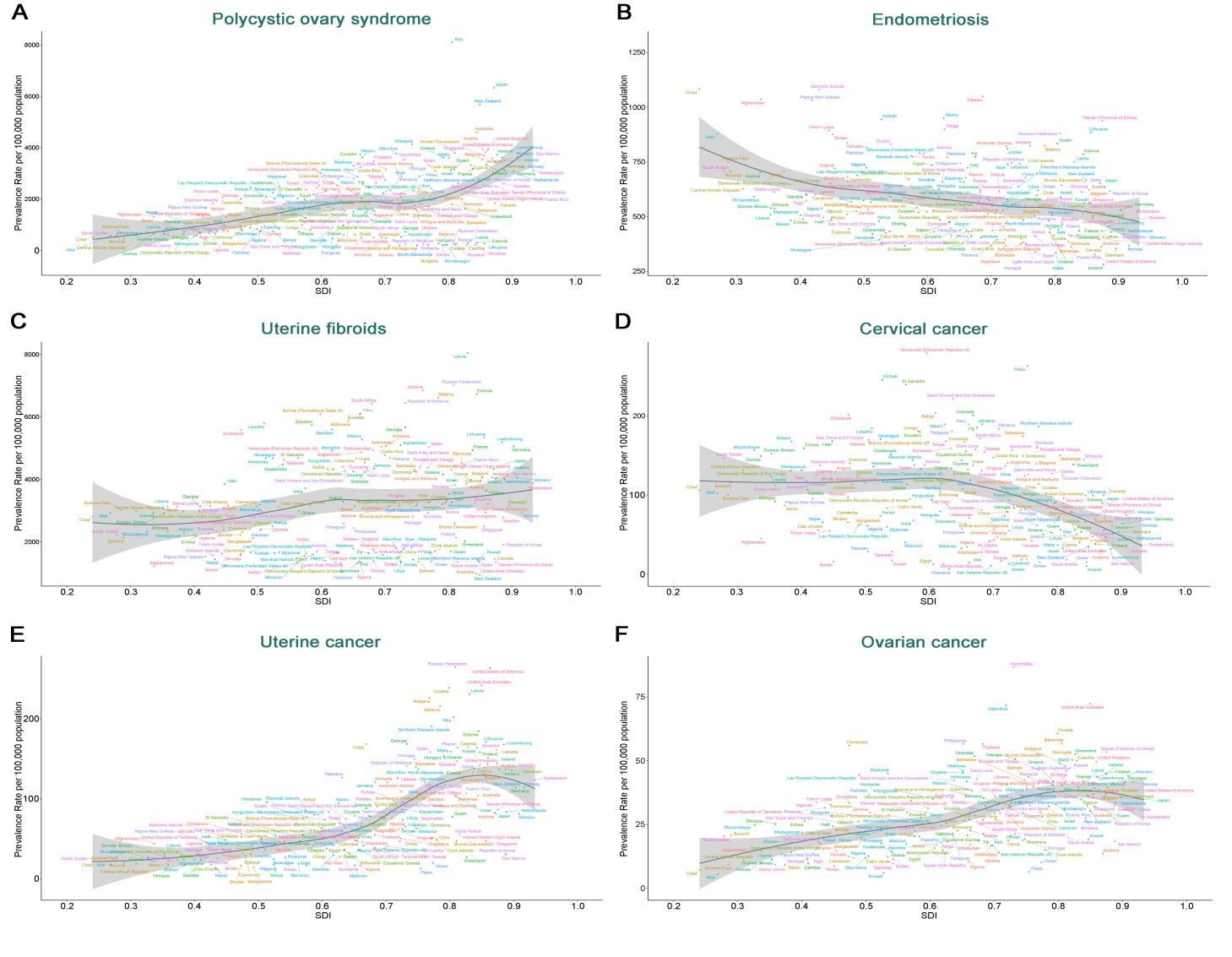


**Figure S11.** ASPR of six gynecological diseases for 204 countries and territories by SDI.


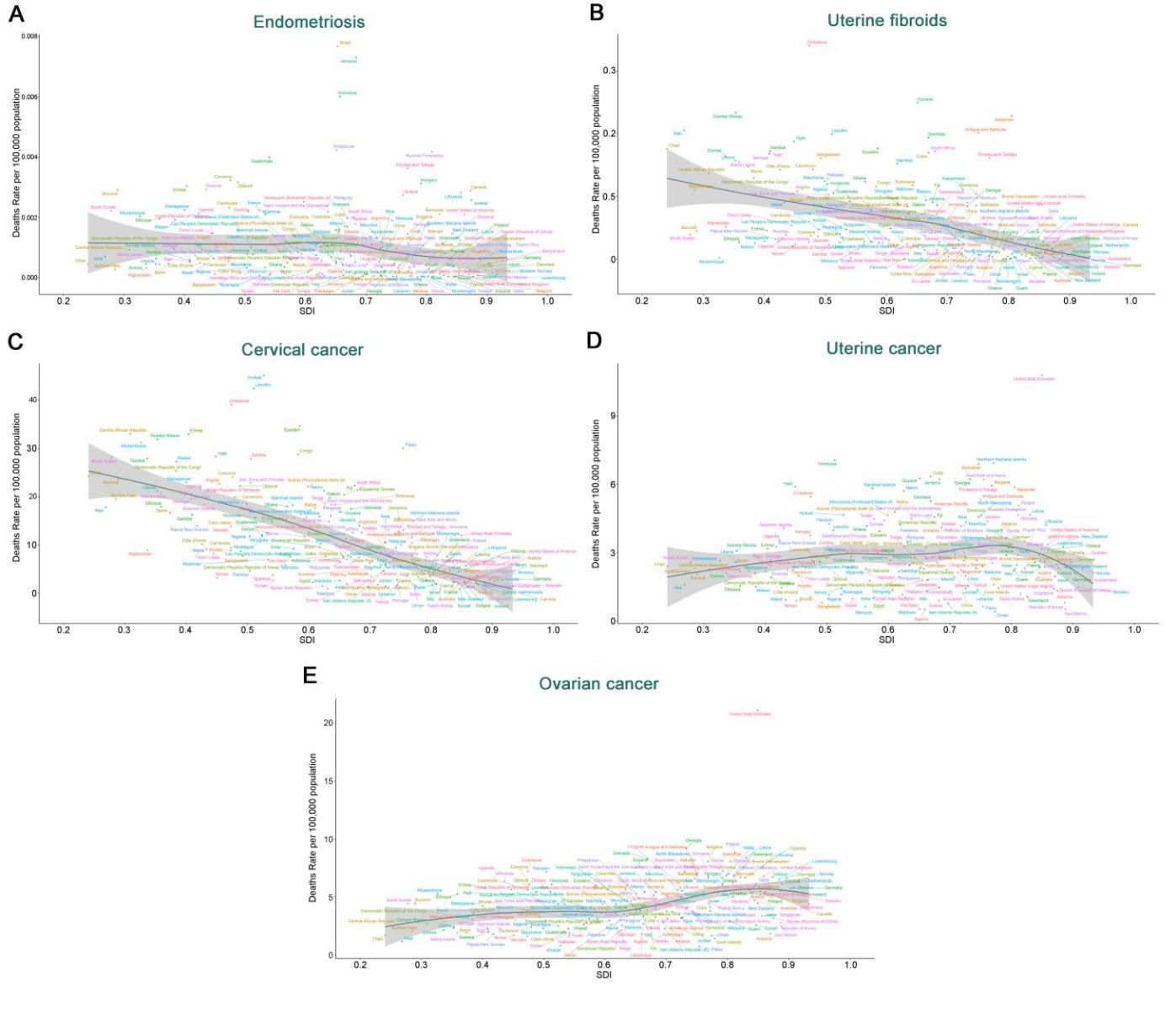


**Figure S12.** ASMR of five gynecological diseases for 204 countries and territories by SDI.


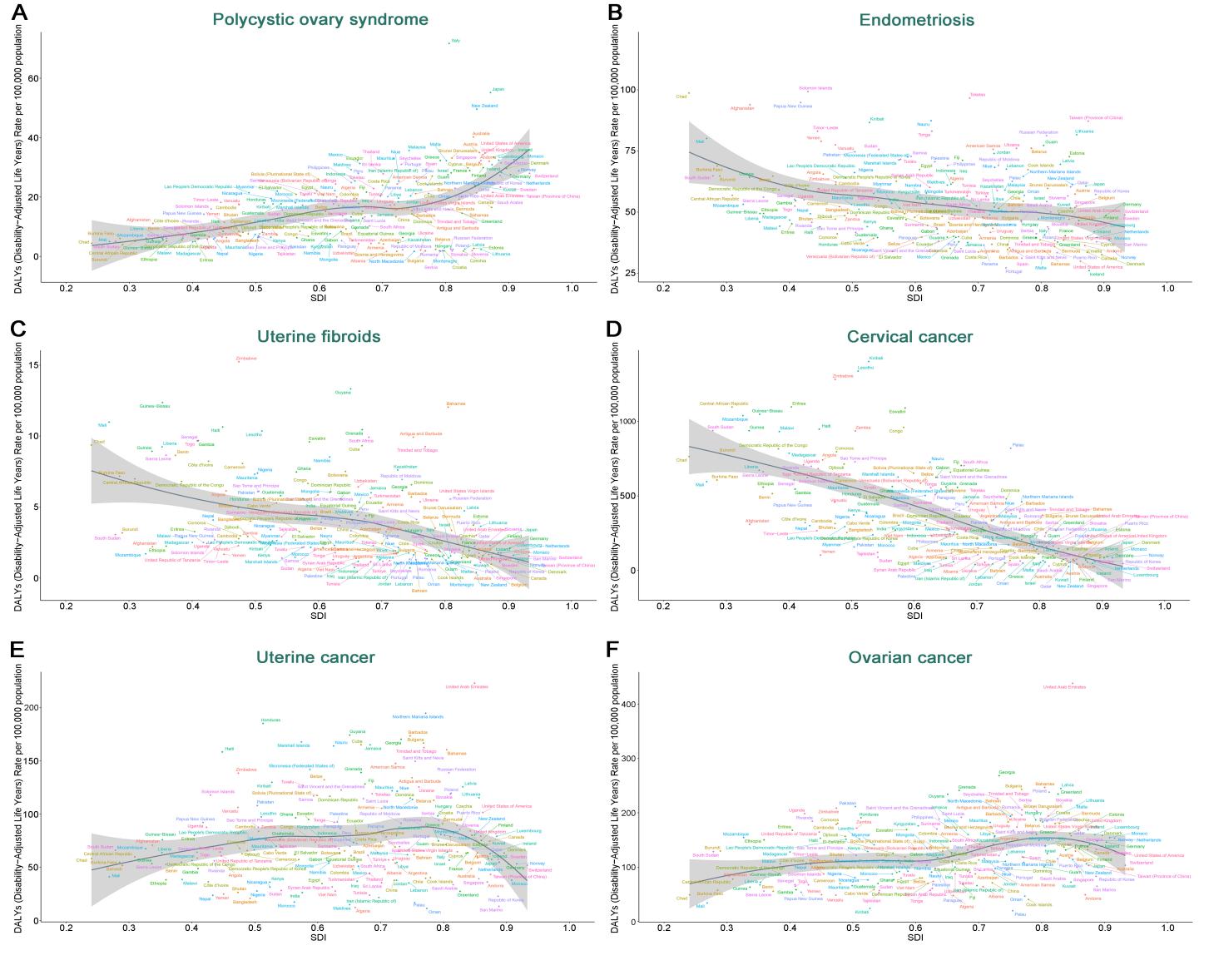


**Figure S13.** ASDR of six gynecological diseases for 204 countries and territories by SDI.


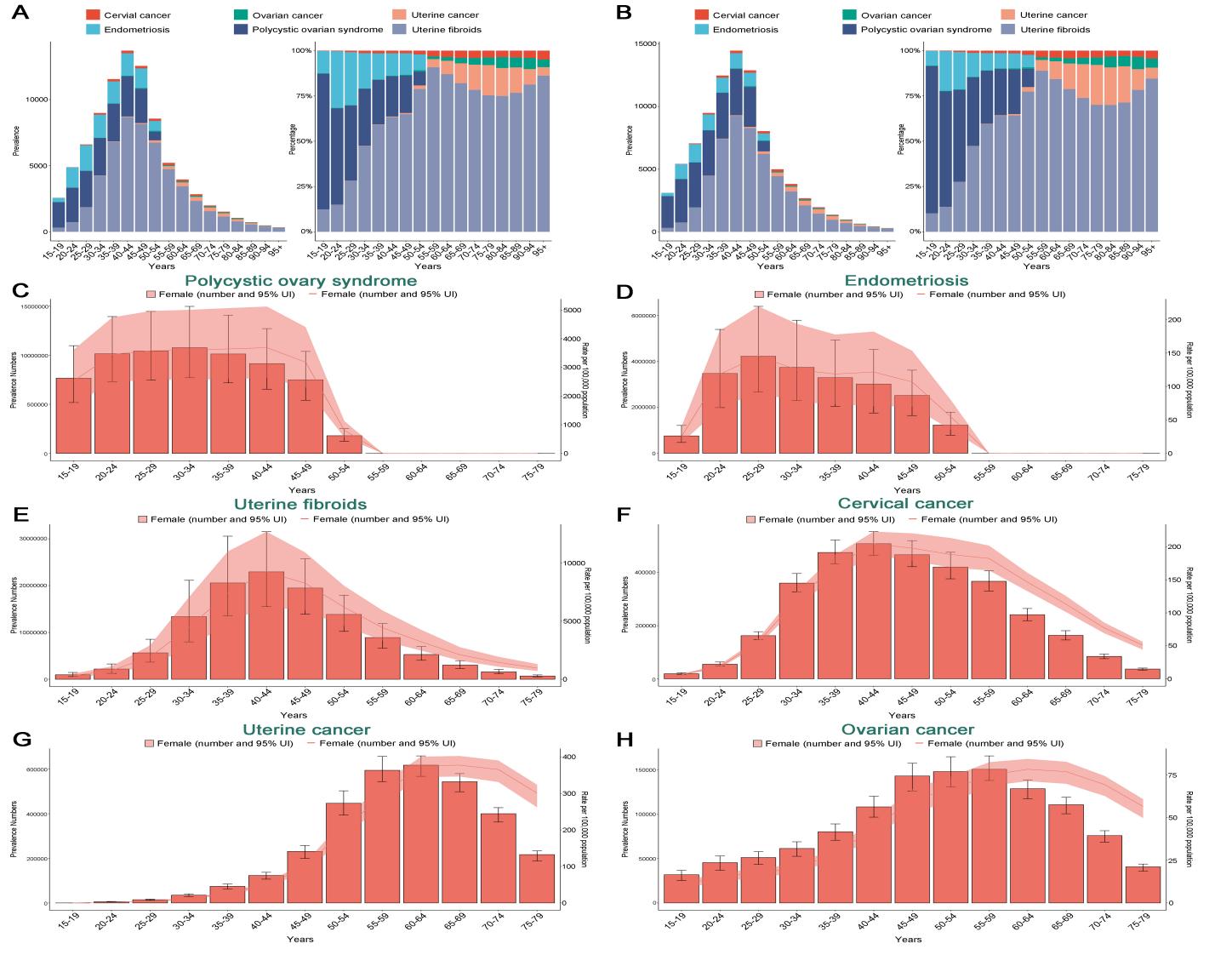


**Figure S14.** Age- and etiology-specific prevalence proportions of gynecological diseases in 1990 and 2021, and the global prevalence of six gynecological diseases by age in 2021.


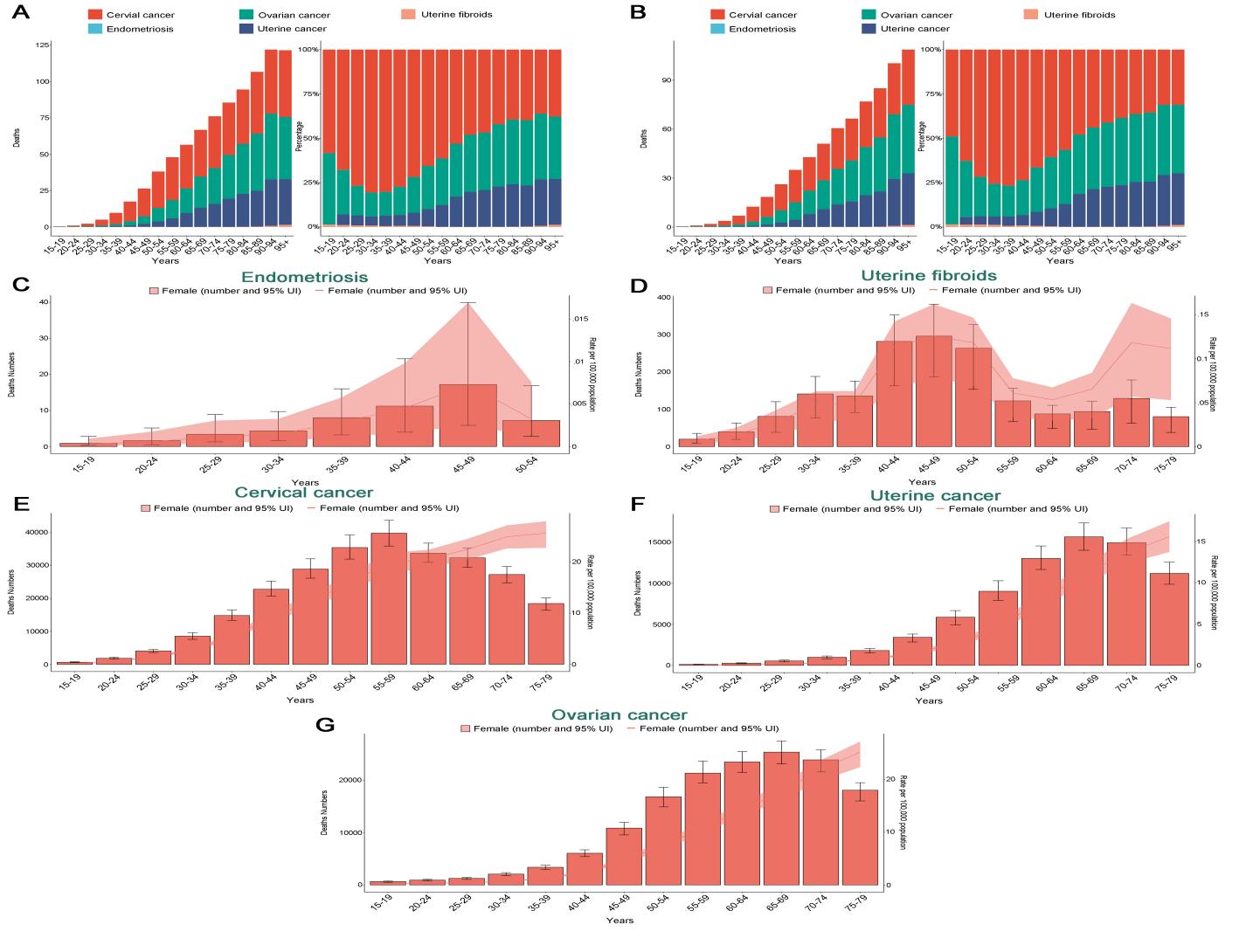


**Figure S15.** Age- and etiology-specific mortality proportions of gynecological diseases in 1990 and 2021, and the global mortality of five gynecological diseases by age in 2021.


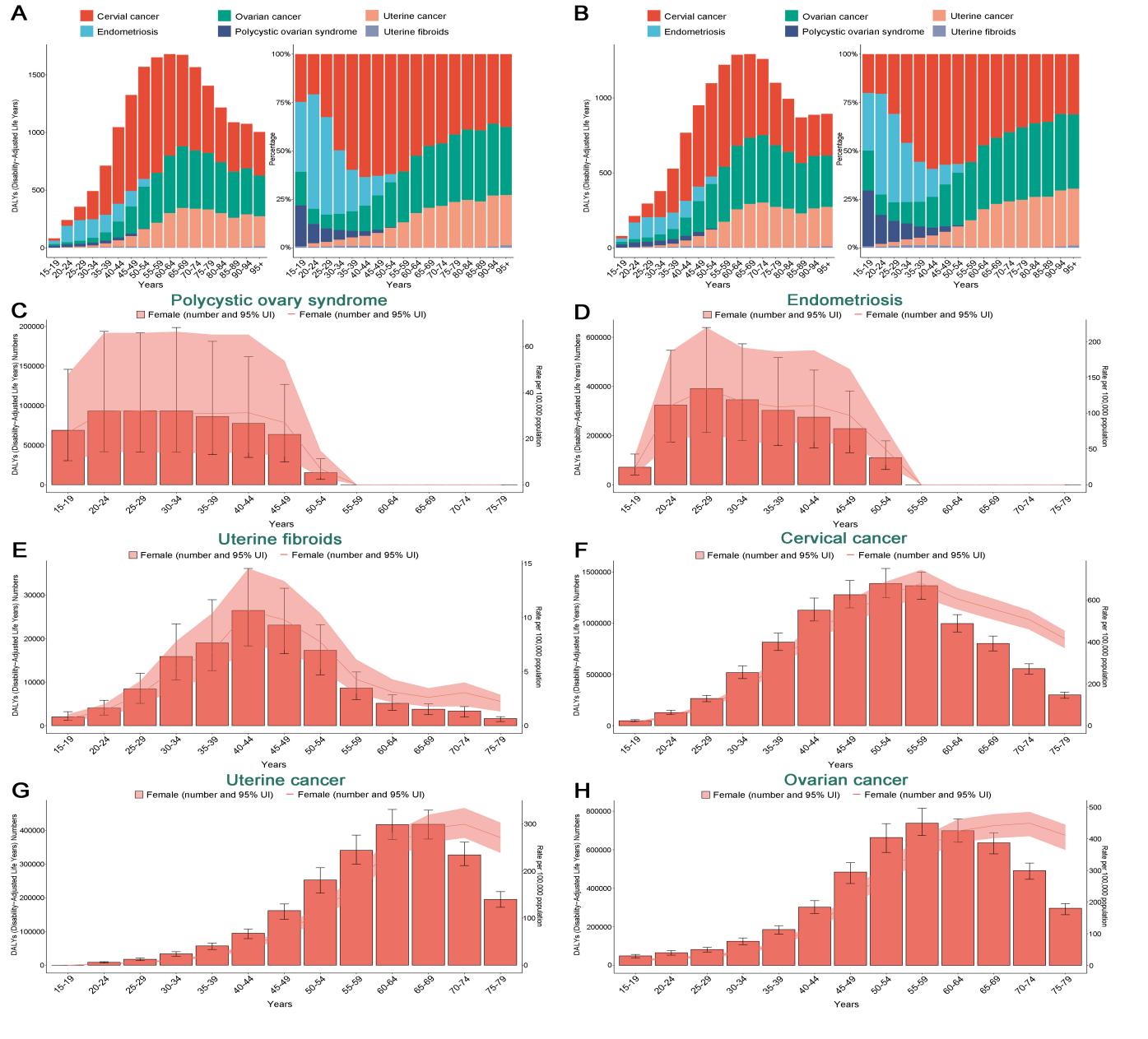


**Figure S16.** Age- and etiology-specific DALYs proportions of gynecological diseases in 1990 and 2021, and the global DALYs of six gynecological diseases by age in 2021.
